# Salivary and Serum Cytokine Concentrations in Kidney Transplantation: A Prospective Study

**DOI:** 10.1111/odi.70012

**Published:** 2025-06-22

**Authors:** Luciano Miziara, Marcio Augusto de Oliveira, Debora Macedo, Ligia Pierrotti, Fabiana Agena, Elias David‐Neto, Aluísio Cotrim Segurado, Rodrigo Zerbinati, Marina Gallottini, Paulo Braz‐Silva, Fabiana Martins

**Affiliations:** ^1^ Postgraduate Program in Dentistry School of Dentistry, University of Santo Amaro São Paulo Brazil; ^2^ Biostatistician São Paulo Brazil; ^3^ Nephrology Service, Hospital das Clínicas HCFMUSP Universidade de Sao Paulo Sao Paulo Brazil; ^4^ Renal Transplant Service, Hospital das Clinicas HCFMUSP Universidade de São Paulo São Paulo Brazil; ^5^ Infectious Diseases Division Medical School, University of São Paulo São Paulo Brazil; ^6^ Laboratory of Virology (LIM‐52), Institute of Tropical Medicine of São Paulo, School of Medicine University of São Paulo São Paulo Brazil; ^7^ Department of Stomatology, School of Dentistry University of Sao Paulo São Paulo Brazil

**Keywords:** acute rejection, clinical outcomes, cytokines, kidney transplantation, saliva

## Abstract

**Background:**

Kidney transplant recipients (KTRs) experience immune modulation, which may lead to graft rejection and other adverse outcomes. Although serum cytokines are well‐established systemic immune markers, the role of salivary biomarkers has never been reported in the literature.

**Objective:**

To investigate salivary and serum cytokine levels in KTRs and their correlations with clinical outcomes over time.

**Materials and Methods:**

We evaluated the same group of 38 KTRs at T1 (< 6 months post‐transplantation) and T2 (> 6 months post‐transplantation). Samples were analysed with Human 6‐Plex Cytokine Panel (Luminex) and clinical data were collected from medical records. Statistical analyses included Wilcoxon tests, Fisher's exact tests, Spearman's correlation, and Benjamini‐Hochberg procedure for multiple comparisons (*p* < 0.05 significant).

**Results:**

Serum cytokines showed lower IFN‐γ levels in cardiac events and associations of TNF‐α, IL‐8, and IL‐10 with cytomegalovirus (CMV), BK polyomavirus (BKPyV) viremia and anaemia. Salivary cytokines showed distinct profiles, with elevated levels of TNF‐α in anaemia and IL‐8 in patients with diarrhoea. Those not experiencing acute rejection in both cases showed reduced salivary IL‐8 levels.

**Conclusions:**

Integrating serum and salivary measurements highlighted the potential of salivary biomarkers, particularly TNF‐α and IL‐8, in complementing traditional blood‐based assays and other invasive monitoring methods in kidney transplantation.

## 1 Introduction

Patients with end‐stage renal disease (ESRD) commonly exhibit an ongoing state of chronic inflammation, associated with uraemia and reduced secretion of anti‐inflammatory cytokines, including impacts of haemodialysis. In this population, pro‐inflammatory cytokines like TNF‐α and IL‐6 are elevated as oxidative stress is induced by the activation of macrophages and monocytes, which perpetuates this inflammatory state (Caglar et al. 2002; Rios et al. 2017).

Several studies have demonstrated that higher serum cytokine levels (e.g., TNF‐α, IL‐6 and IL‐8) in kidney transplant recipients (KTRs) are correlated with adverse clinical outcomes, such as chronic rejection (CR) and acute rejection (AR) (Alves et al. 2020; Mota et al. 2013).

The immune response changes significantly over time following a kidney transplantation, which reflects the dynamic equilibrium between immunosuppression and immune activation. Acute immune activation and increased rejection risk are related to the immune pro‐inflammatory profile in the early post‐transplantation phase (typically within the first year), which is characterized by increased expression of cytokines like IL‐2, IFN‐γ, and IL‐6. As time progresses in the long‐term period (i.e., beyond the first year), the cytokine profile shifts towards playing a more chronic regulatory injury‐related role, marked by increased levels of IL‐10, TGF‐β, IL‐17, and IL‐21. The long‐term impacts of graft function and survival are affected by this transition, which is the result of a complex interaction between persistent alloimmune responses, chronic inflammation, and immunomodulatory effects of prolonged immunosuppressive therapy. (Mota et al. 2013; Hribova et al. 2005; Li and Lan 2023).

Authors suggest that monitoring the post‐transplantation levels of cytokine can be used for assessment of immune function, resulting in personalised immunosuppressive therapy and improved outcomes (Alves et al. 2020; Mota et al. 2013; Sonkar et al. 2013; Møller et al. 2023; Dongiovanni et al. 2023).

Saliva, a multipurpose fluid, is attracting interest as a potential diagnostic tool due to its non‐invasive collection technique requiring no specialised personnel (Dongiovanni et al. 2023; De Santana Sarmento et al. 2017). Despite an extensive documentation of the correlation between cytokine quantification in serum, the comparison between these two biological fluids is not reported in the current literature.

Therefore, this study aimed to measure and correlate cytokine levels in saliva and serum of KTRs by monitoring their concentrations in relation to clinical outcomes over time.

## 2 Materials and Methods

This longitudinal observational study aimed to follow up a group of KTRs (> 18 years) at two times after transplantation as follows: T1 (less than 6 months post‐transplantation) and T2 (more than 6 months post‐transplantation). Patients were recruited based on the requirements outlined in an approved institutional review board protocol (IRB# 90602418.4.0000.0068).

The search for patients was conducted by using the hospital's electronic medical records system for identification of individuals who met the inclusion criteria based on their scheduled consultations. The initial screening consisted of filtering records by appointment dates within the study period. The eligibility of each patient was evaluated through a review of his or her records, which contained clinical, demographic, and procedural information.

All the participants selected from a Brazilian transplantation centre read and signed an informed consent form, indicating their willingness to participate in the study. Participants were included during the period from August 2019 to January 2023. Patients admitted to the intensive care unit (ICU) were excluded from the study.

To minimise potential fluctuations in cytokine levels due to circadian or dietary influences, both peripheral venous blood and saliva samples were collected in the early fasting state during routine post‐transplant evaluations. Blood was drawn from each participant by a research nurse using standard venipuncture techniques and placed into pre‐labelled dry tubes. For saliva collection, researchers used the Salivette test kit (Sarstedt S.A.), which consists of a tube and a cotton roll. Patients first rinsed their mouths with distilled water for 1 min, after which the cotton roll was positioned on the floor of the mouth for 5 min to ensure thorough moisture saturation. All samples were immediately stored at −20°C in a cooler and then sent to the laboratory, where they were centrifuged (Centrifuge 5810, Eppendorf) at 4.000 rpm for 5 min and subsequently stored at −80°C until total nucleic acid extraction (De Santana Sarmento et al. 2017). The cytokine concentrations from all patients included in the study were analysed by using the Cytokine Human 6‐Plex Panel (Luminex Corporation, Austin, TX, USA), according to the manufacturer's instructions, for quantifying the following cytokines: IL‐6, IFN‐γ, IL‐10, IL‐8, IL‐4, and TNF‐α.

Following sample collection, all the enrolled patients underwent an intraoral clinical evaluation at T1. These examinations were carried out by three expert dentists who are familiar with identifying and evaluating oral alterations, infectious foci, or medication‐related toxicities. Immediately after the oral examination, all clinical results were inserted into standardized forms.

The researchers conducted a manual search of all electronic medical records for gathering demographic data, clinical outcomes, laboratory tests, details of immunosuppressive therapy, side effects of therapy, chronic or acute rejection, and opportunistic infections (OI). All clinical data were collected from T1 to T2.

The primary aim of the statistical analyses was to compare cytokine levels in saliva and blood over time at T1 and T2, as well as clinical parameters (e.g., CMV and BKPyV viremia), laboratory findings, adverse events probably associated with the immunosuppressive regimen, cardiovascular events, other infectious occurrences, hypersensitivity, mortality rate, and acute or chronic rejection (AR, CR respectively).

JMP Pro software (version 13, SAS Institute Inc., Cary, NC, USA) was used for statistical tests, in which non‐parametric Wilcoxon test for continuous measurements with non‐normal distribution and Fisher's exact test for comparison between percentages were applied at a significance level of 0.05 (95% confidence). For correlations between saliva and serum, we used Spearman's correlation test (Fleiss and Agresti 1991).

**FIGURE 1:**
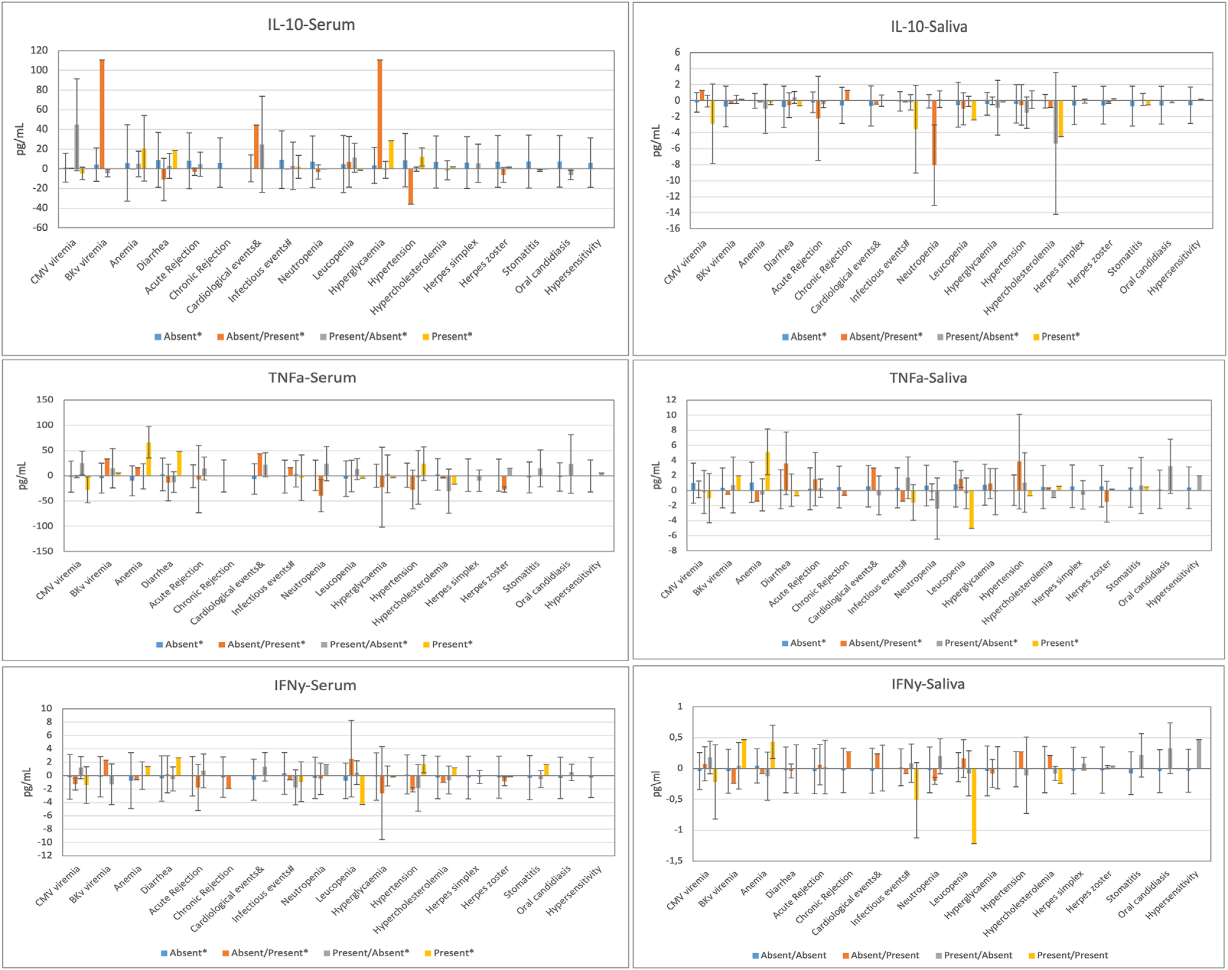
Comparison of cytokine levels with time and clinical alterations (Wilcoxon non‐parametric test).

Benjamini‐Hochberg procedure was used to control the false discovery rate (FDR) at a level of 0.05 in order to account for multiple comparisons in the cytokine analyses, thus ensuring robust identification of significant associations while minimising false positives (Benjamini and Hochberg 1995).

## 3 Results

We initially enrolled 54 adult KTRs in this study. However, six patients died before the first time point (T1), thus leaving 48 participants for full biological sampling. Moreover, we lost contact with five participants and another five had died at the second time point (T2), resulting in 38 patients completing evaluations at T2. The mean follow‐up time at T1 was 5.89 ± 1.13 months, whereas at T2 it was 32.9 ± 6.1 months.

Among the 48 participants, 52.1% were female and 47.9% were male, with a mean age of 51.9 years (±13.1). Most participants were receiving immunosuppressive regimens that included cyclosporine (Cy) or tacrolimus (FK), mTOR inhibitors (EVL), azathioprine (AZA), and corticosteroids (Pred). Detailed demographic information is provided in Table 1.

**TABLE 1:** Demographics and clinical characteristics of the study cohort (*n* = 48).

Age years [mean ± SD]	51.9 [51.9 ± 13.1]
Female	52.1
Male	47.9
Type of donor	
Deceased	46 (95.8%)
Living	2 (4.2%)
Immunosuppressive therapy	
Prednisone/Tacrolimus/Everolimus	14 (29.8%)
Prednisone/Tacrolimus/MMF	15 (31.9%)
Prednisone/Tacrolimus/MPS	13 (27.7%)
Prednisone/Everolimus	3 (6.4%)
Prednisone/Tacrolimus/Azathioprine	2 (4.3%)
Pre‐transplant disease	
Hypertension	19 (39.6%)
Diabetes mellitus	13 (27.1%)
Autoimmune	6 (12.5%)
Others	10 (13%)
CMV serostatus [*n* (%)]	46 (98%)
Haemodialysis	45 (93%)
Time on dialysis (months)	76.8 ± 59.9

### 3.1 Clinical Outcomes

Table 2 presents the frequencies of clinical changes observed in the 38 patients assessed at both T1 and T2. Several outcomes showed statistically significant improvements over time, namely: BKPyV viremia decreased from 22.9% to 5.3% (*p* = 0.0339), anaemia declined from 54.2% to 7.9% (*p* < 0.0001), infectious events decreased from 39.5% to 13.2% (*p* = 0.0039), hyperglycaemia decreased from 31.3% to 7.9% (*p* = 0.0027), oral herpes was reduced from 10.4% to 0% (*p* = 0.0455), oral candidiasis fell from 10.4% to 0% (*p* = 0.0253), and stomatitis decreased from 25.0% to 2.6% (*p* = 0.0027).

**TABLE 2:** Prevalence of clinical changes over time using Bowker's symmetry test (*p* < 0.05).

		Present	Absent	*p*
CMV viremia	T1	18 (37.5%)	30 (62.5%)	0.1317
	T2	8 (21.1%)	30 (78.9%)	
BKPyV viremia	T1	11 (22.9%)	37 (77.1%)	0.0339
	T2	2 (5.3%)	36 (94.7%)	
Anaemia	T1	26 (54.2%)	22 (45.8%)	< 0.0001
	T2	3 (7.9%)	35 (92.1%)	
Diarrhoea	T1	9 (18.8%)	39 (81.3%)	0.1317
	T2	4 (10.5%)	34 (89.5%)	
Acute rejection	T1	4 (10.5%)	34 (89.5%)	0.7389
	T2	5 (13.2%)	33 (86.8%)	
Chronic rejection	T1	0 (0.0%)	38 (100.0%)	0.3173
	T2	1 (2.6%)	37 (97.4%)	
Cardiovascular events^a^	T1	7 (18.4%)	31 (81.6%)	0.0956
	T2	2 (5.3%)	36 (94.7%)	
Infectious events^b^	T1	15 (39.5%)	23 (60.5%)	0.0039
	T2	5 (13.2%)	33 (86.8%)	
Neutropenia	T1	2 (5.3%)	36 (94.7%)	1.0000
	T2	2 (5.3%)	36 (94.7%)	
Leukopenia	T1	12 (25.0%)	36 (75.0%)	0.1967
	T2	6 (15.8%)	32 (84.2%)	
Hyperglycaemia	T1	15 (31.3%)	33 (68.8%)	0.0027
	T2	3 (7.9%)	35 (92.1%)	
Hypertension	T1	8 (21.1%)	30 (78.9%)	0.1573
	T2	4 (10.5%)	34 (89.5%)	
Hypercholesterolemia	T1	3 (7.9%)	35 (92.1%)	0.5637
	T2	2 (5.3%)	36 (94.7%)	
Herpes simplex	T1	5 (10.4%)	43 (89.6%)	0.0455
	T2	0 (0.0%)	38 (100.0%)	
Herpes zoster	T1	1 (2.1%)	47 (97.9%)	0.5637
	T2	2 (5.3%)	36 (94.7%)	
Stomatitis	T1	12 (25.0%)	36 (75.0%)	0.0027
	T2	1 (2.6%)	37 (97.4%)	
Oral candidiasis	T1	5 (10.4%)	43 (89.6%)	0.0253
	T2	0 (0.0%)	38 (100.0%)	
Hypersensitivity	T1	2 (4.2%)	46 (95.8%)	0.1573
	T2	0 (0.0%)	38 (100.0%)	

^a^ Cardiovascular events: thrombophlebitis; atherosclerosis; atherosclerosis; chest pain; deep vein thrombosis; renal artery stenosis.

^b^ Infectious events: *E. faecalis*, pseudomonas, pneumonia, urinary tract infection— *E. faecium* resistant to vancomycin, staphylococcus aureus, sepsis, candida glabrata, *Candida albicans* , acute tubular necrosis, subcutaneous seroma, COVID, flu.

### 3.2 Cytokines Assessment

To account for multiple comparisons, *p*‐values from cytokine analyses were adjusted by using the Benjamini‐Hochberg adjustment procedure for control of the false discovery rate (FDR) at 0.05. Of the 19 tested associations, 15 remained statistically significant after such adjustment.

IFN‐γ (serum): Analysis of serum levels of IFN‐γ revealed a significant difference between patients who experienced cardiac events (CE) and those who did not at T1. Specifically, patients who had CE exhibited significantly lower serum levels of IFN‐γ (*p* = 0.0392).

TNF‐α (serum): Significant differences were observed at both time points. At T1, patients who developed stomatitis had significantly lower serum levels of TNF‐α compared to those without stomatitis (*p* = 0.0351). At T2, patients negative for CMV infection showed significantly higher serum levels of TNF‐α compared to those with CMV infection (*p* = 0.0398). Conversely, patients who remained CMV‐positive at both times exhibited lower TNF‐α concentrations (*p* = 0.0153). Serum TNF‐α levels were also elevated in patients without anaemia compared to those with anaemia at T2 (*p* = 0.0133).

TNF‐α (saliva): High levels of TNF‐α were significant in patients presenting anemia at both times (*p* = 0.0329).

IL‐10 (serum): Patients who developed hyperglycaemia at T2 had significantly higher serum levels of IL‐10 compared to those without hyperglycaemia (*p* = 0.0314). Patients transitioning from CMV‐positive to CMV‐negative status (T1/T2) exhibited increased IL‐10 levels (*p* = 0.0360), as did those who developed BKPyV infection at T2 (*p* = 0.0298).

IL‐8 (serum): At T2, patients negative for CMV infection exhibited significantly higher serum levels of IL‐8 compared to infected ones (*p* = 0.0345). Patients transitioning from CMV‐positive to CMV‐negative status (T1/T2) also had elevated IL‐8 levels (*p* = 0.0274).

IL‐8 (saliva): Analysis of IL‐8 concentrations at T2 revealed significant differences in patients who experienced diarrhoea, as they showed significantly higher salivary concentrations of IL‐8 compared to those without this adverse event (*p* = 0.0087). When examining cytokine evolution, patients with persistent diarrhoea across both time points showed an increase in salivary IL‐8 levels (*p* = 0.0379). In contrast, herpes simplex infection at T1 was associated with lower IL‐8 levels (*p* = 0.0308). Patients who remained free from acute rejection at both times exhibited lower IL‐8 levels (*p* = 0.0254) (Figure 1).

**FIGURE 2:**
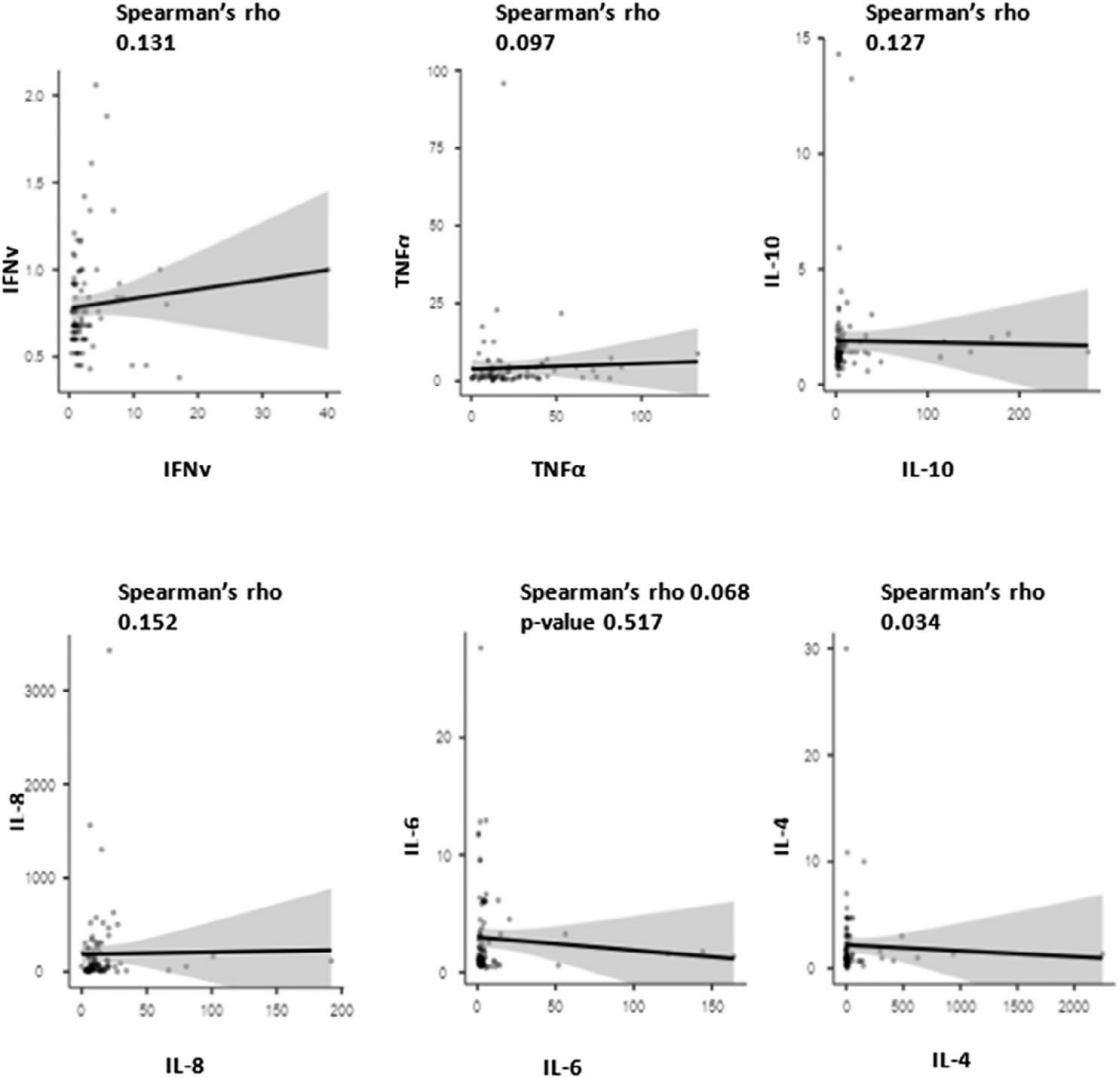
The Spearman correlation test was employed to assess the potential direct relationship between serum and saliva cytokine levels. None of the cytokine assays exhibited a direct correlation.

Additionally, we analysed and compared the two collection methods (i.e., serum and saliva) by using Spearman's correlation test, with the following values being considered: 0.00 to 0.19 ‘very weak’; 0.20 to 0.39 ‘weak’; 0.40 to 0.59 ‘moderate’; 0.60 to 0.79 ‘strong’; and 0.80 to 1.0 ‘very strong’. We considered that correlations between the two methods were very weak or weak when there was a direct relationship between them without statistical significance (Figure 2).

## 4 Discussion

This study aimed to investigate the correlation between a specific set of cytokines and adverse clinical outcomes, such as infections and oral changes, in patients undergoing kidney transplantation. To date, no studies in the literature have compared these biomarkers in blood and especially in saliva for the observation of their progression over time in a cohort of KTRs.

Several studies have evaluated the role of serum cytokine expression before and after solid organ transplantation by focusing on both short‐term and long‐term immunological responses. However, there is a notable lack of research specifically assessing adverse clinical outcomes by using saliva samples from kidney transplant recipients, as most existing studies have been conducted in patients with end‐stage renal disease rather than in post‐transplant populations. (Mota et al. 2013; Møller et al. 2023; De Santana Sarmento et al. 2017).

According to some authors, assessing serum cytokine levels can help identify underlying diseases that lead to acute or chronic rejection, two of the leading causes of mortality and morbidity among kidney transplant recipients. Although pre‐transplant levels of IL‐6, IL‐10, and IFN‐γ have been found to be predictors of subsequent organ failure, several studies have demonstrated that elevated serum levels of IFN‐γ, IL‐17, and IL‐2 are linked to a higher risk of AR in patients receiving liver and kidney transplants (Sonkar et al. 2013; Heldal et al. 2022).

To ensure robust statistical inference given the multiple comparisons in our study (approximately 50–60 tests), we applied the Benjamini‐Hochberg procedure for control of the false discovery rate (FDR). Of the 19 *P*‐values tested for cytokine associations, 15 remained statistically significant after such adjustment, thus reinforcing the reliability of our findings while balancing the risk of false positives (Benjamini and Hochberg 1995).

In the present study, patients who experienced CE demonstrated significantly lower serum levels of IFN‐γ at T1. This finding corresponds to the recognized function of IFN‐γ in stimulating macrophages and enhancing Th1 responses, which are essential for efficient immune surveillance and pathogen elimination (Halloran et al. 2001).

In the context of KTRs, IFN‐γ played dual roles as it enhances the expression of major histocompatibility complex molecules and supports immune monitoring, while also preserving graft integrity by preventing early vascular damage. Research using mouse models has shown that the lack of IFN‐γ receptors in transplanted kidneys results in increased thrombosis and necrosis, highlighting its significance in maintaining graft function. From a clinical standpoint, the role of IFN‐γ has been implicated in AR events resulting in inflammation, which may indirectly contribute to cardiovascular problems (Halloran et al. 2001; Molnar et al. 2017; Zhou et al. 2015; Boix et al. 2017).

Pro‐inflammatory cytokines TNF‐α and IL‐6 are also implicated in cardiac events (CE). TNF‐α reduces endothelial nitric oxide synthase activity, promoting reactive oxygen species, while IL‐6 drives atherosclerosis via adhesion molecule and chemokine up‐regulation (Halloran et al. 2001; Boix et al. 2017). Despite the observation of elevated serum levels of TNF‐α in patients with CE at T2, these findings failed to maintain statistical significance following FDR adjustment. This correlation was not observed in IL‐6 concentrations.

Significant differences in TNF‐α serum levels were noted in individuals who had stomatitis associated with imTOR inhibitors, CMV viremia, and anaemia. TNF‐α is a pro‐inflammatory cytokine present in systemic inflammation and the acute phase reaction after transplantation. Lower TNF‐α levels in these conditions may reflect a compromised inflammatory response, which could predispose patients to infections and clinical adverse events (Mota et al. 2013).

mTOR inhibitors lead to stomatitis, or mIAS, which is characterized by oral ulcerations with aphthous‐like lesions predominantly affecting the movable oral mucosa with rapid onset. (Martins et al. 2013; Sonis et al. 2016) Among the 26 patients receiving everolimus, 10 cases of mIAS were identified at T1. Sonis and collaborators used an organotypic model to elucidate the pathogenesis of mIAS, finding that exposure to everolimus led to epithelial injury, increased apoptosis, and decreased proliferation, accompanied by elevated levels of IL‐6, IL‐8, and IFN‐γ (Sonis et al. 2016). However, the present investigation did not identify this association, although the TNF‐α level was lower. This discrepancy may highlight a tissue‐specific cytokine dynamics.

CMV reactivation is common in KTRs and is frequently related to immunosuppression. Cytokines like TNF‐α can influence the severity and duration of CMV infection. Elevated TNF‐α levels, in conjunction with IFN‐γ, may facilitate the immune response against CMV, but excessive inflammation can lead to tissue damage and adverse outcomes, including graft rejection (Zhou et al. 2015). In the present investigation, patients who tested positive for CMV at both time points demonstrated reduced TNF‐α values, thus corroborating this association (Boix et al. 2017; Sadeghi et al. 2008).

Anaemia is a common finding in both chronic renal disease patients and KTRs, as it is linked to TNF‐α‐mediated inhibition of erythropoiesis and iron metabolism (Halloran et al. 2001). Our findings revealed elevated serum levels of TNF‐α in non‐anaemic patients and increased salivary levels of TNF‐α in anaemic patients at both time points. This systemic‐local dichotomy suggests distinct roles played by TNF‐α in KTRs with anaemia, with salivary levels potentially reflecting localised mucosal inflammation (Nemeth and Ganz 2014).

TNF‐α also plays a key role in inducing insulin resistance, which is recognised as a predictor of mortality and poor outcomes in KTRs. Insulin resistance and associated metabolic disturbances can further impair erythropoiesis and contribute to the development of anaemia. Interestingly, although elevated TNF‐α levels associated with anaemia were observed in our study, there was no significant relationship between TNF‐α and hyperglycaemia, possibly due to Benjamini‐Hochberg adjustment for filtering out weaker effects. Also, hyperglycaemic patients exhibited significantly elevated IL‐10 levels, suggesting that anti‐inflammatory or compensatory immune regulatory pathways may contribute to metabolic changes observed in this cohort of KTRs (Molnar et al. 2017; Nemeth and Ganz 2014).

Serum IL‐10 levels during hyperglycaemia can counteract the pro‐inflammatory state due to inflammation, immunosuppressive therapy effects, and metabolic changes associated with diabetes. The complex relationship between immune function and metabolic health is observed in the KTR population. Some authors found a correlation between decreased levels of IFN‐γ levels and increased levels of IL‐10, associated with an increased risk of oral candidiasis in diabetic patients, but this relationship was not observed in the present study. (Carlini et al. 2023; Halimi et al. 2022) In our study, there were significant associations between higher serum titrations of IL‐10 and opportunistic infections, such as CMV and BKPyV viremia at T2.

Mota and colleagues reported that shortly after transplantation, the circulating levels of regulatory cytokines (particularly IL‐4, IL‐5 and IL‐10) are prevalent, possibly due to their association with immunosuppression resulting from induction therapy. This anti‐inflammatory environment is desirable as it can promote tolerance and improve graft survival (Mota et al. 2013). On the other hand, elevated IL‐10 levels can increase susceptibility to opportunistic infections by inhibiting immune activation.

In contrast to other cytokines, studies show that the levels of IL‐10 are maintained high or stable, indicating a boost in the anti‐inflammatory response following transplantation. BKPyV viremia typically manifests during extended intervals after transplantation. Other studies evaluating IL‐10 polymorphisms found that the rs1800872 TT genotype was associated with an increased risk and earlier occurrence of BKPyV viremia after kidney transplantation, indicating that host genetic factors interact to modulate the risk of BKPyV infection following transplantation (Mota et al. 2013; Li and Lan 2023; Møller et al. 2023; Redondo et al. 2022).

The elevated serum levels of IL‐8 at T2 were observed in negative cases for CMV, in which a tendency for BKPyV viremia and absence of neutropenia may indicate a clinical immunological response commonly associated with long‐term transplantation as some patients were evaluated 12 months after transplantation. Sonkar and colleagues describe that pro‐inflammatory cytokines are elevated in KTRs, triggering an immune response mediated by TH1 and TH2 lymphocytes (Sonkar et al. 2013). These findings suggest that serum IL‐8 may be associated with the immune response to viral infections, exhibiting a unique pattern of cytokine change based on the presence or absence of viruses. The same pattern was observed in cases of high salivary levels of IL‐8, indicating an active inflammatory response to infections (e.g., herpes *labialis*) or adverse effects of the immunosuppression therapy (e.g., diarrhoea).

Literature has investigated the potential of IL‐8 levels as a biomarker for AR in KTRs. It has been shown that urinary IL‐8 concentrations are elevated in patients who experience biopsy‐proven AR within the first few months after transplantation. However, this relationship was not observed in serum samples (Møller et al. 2023; García‐Covarrubias et al. 2020). The present study found a connection between individuals testing negative for AR and lower levels of IL‐8 in saliva samples. Benjamini‐Hochberg adjustment strengthens the reliability of these findings on IL‐8, thus highlighting the saliva's potential as a non‐invasive diagnostic medium for monitoring systemic outcomes in KTRs.

The use of saliva as an alternative diagnostic method is suggested due to its non‐invasive nature and cost‐effectiveness for the collection and storage of substantial sample volumes. Consequently, the ability to identify and measure biomarkers in saliva samples is increasingly attractive for research and clinical applications focused on the early diagnosis of chronic diseases and the monitoring of ongoing disease. (Dongiovanni et al. 2023; De Santana Sarmento et al. 2017).

Although some studies have verified an increase in salivary cytokines in different chronic diseases, none have examined KTR‐correlated cytokine levels in both blood and saliva. In this study, the correlation between the two fluids was weak or very weak, except for the levels of INF‐y, whose salivary and serum levels were significantly correlated.

An important strength of this study is its longitudinal design, as few investigations have followed up the same group of kidney transplant recipients over time to evaluate oral health outcomes and inflammatory marker dynamics. Most previous studies have focused on serum cytokine profiles, providing valuable insights into systemic inflammation but offering little information about the local oral environment. To our knowledge, this is one of the first studies to integrate salivary cytokine measurements in a longitudinal cohort of KTRs, thus providing a novel perspective on local inflammatory processes that may contribute to oral disease in these patients.

While this study provides important insights, certain limitations should be recognised. The follow‐up period, despite encompassing two time points, may inadequately reflect long‐term trends or delayed effects in salivary cytokine dynamics and clinical outcomes. Furthermore, although the sample size was sufficient for identifying within‐subject changes, it restricts the power for subgroup analyses. Due to the sanitary restrictions imposed during the SARS‐CoV‐2 pandemic, the follow‐up duration was not feasible. However, the study successfully monitored and evaluated the clinical and immunological aspects of the same patient at two distinct time points, approximately 1 year following kidney transplantation. Salivary cytokine levels may also be affected by local oral factors, including salivary flow, shifts in microbiota, and oral hygiene, which were not completely standardised or controlled, potentially leading to variability. Future research should include extended follow‐up periods and a larger population.

## 5 Conclusions

In conclusion, this study provides novel insights into the dynamic interplay between systemic and salivary cytokine profiles and their associations with adverse clinical outcomes, infections, and oral changes in kidney transplant recipients. By integrating serum and saliva measurements longitudinally, we highlight the potential of non‐invasive salivary biomarkers, particularly IFN‐γ and IL‐8, to complement the traditional blood‐based monitoring. Despite limitations such as sample size, follow‐up duration, and uncontrolled oral factors, our findings emphasize the importance of further research with larger cohorts and extended observation time to validate these associations and improve post‐transplant surveillance and patient care.

## Author Contributions

**Luciano Miziara:** investigation, data curation, writing – original draft. **Marcio Augusto de Oliveira:** formal analysis, writing – original draft, visualization. **Debora Macedo:** investigation, data curation, project administration, visualization. **Ligia Pierrotti:** conceptualization, supervision. **Fabiana Agena:** project administration. **Elias David‐Neto:** supervision. **Aluísio Cotrim Segurado:** supervision. **Rodrigo Zerbinati:** data curation, investigation, validation. **Marina Gallottini:** supervision, resources. **Paulo Braz‐Silva:** conceptualization, methodology, resources. **Fabiana Martins:** writing – review and editing, supervision, conceptualization, funding acquisition, project administration, visualization, resources, investigation.

## Conflicts of Interest

The authors declare no conflicts of interest.

## Supporting information


**Table S1.** Descriptive means and standard deviation for cytokines and clinical outcomes at T1 (nonparametric Wilcoxon test; *p* < 0.05).
**Table S2.** Descriptive means and standard deviation for cytokines and clinical outcomes at T2 (nonparametric Wilcoxon test; *p* < 0.05).
**Table S3.** Evolution of cytokines through the progression of patient characteristics (nonparametric Wilcoxon test; *p* < 0.05).

## Data Availability

The data that support the findings of this study are available on request from the corresponding author. The data are not publicly available due to privacy or ethical restrictions.
