# Supplementary material for: Salivary and Serum Cytokine Concentrations in Kidney Transplantation: A Prospective Study
Source: Oral Dis. 2025 Jun 22;31(12):3474–81. doi: 10.1111/odi.70012 (PMC12989047; doi:10.1111/odi.70012)
Supplement: Supplementary file 1 — Table S1. Descriptive means and standard deviation for cytokines and clinical outcomes at T1 (nonparametric Wilcoxon test; p < 0.05). Table S2. Descriptive means and standard deviation for cytokines and clinical outcomes at T2 (nonparametric Wilcoxon test; p < 0.05). Table S3. Evolution of cytokines through the progression of patient characteristics (nonparametric Wilcoxon test; p < 0.05). [file ODI-31-3474-s001.docx]

**Supplementary Table 1.**  Descriptive means and standard deviation for cytokines and clinical outcomes at T1 (nonparametric Wilcoxon test; p-values < 0.05)

**Supplementary Table 2.**  Descriptive means and standard deviation for cytokines and clinical outcomes at T2 (nonparametric Wilcoxon test; p-values < 0.05)

**Supplementary Table 3.** Evolution of cytokines through the progression of patient characteristics (nonparametric Wilcoxon test; p-values < 0.05)

**Supplementary Table 1:** Descriptive means and standard deviation for cytokines and clinical outcomes at T1 (*nonparametric Wilcoxon test)*

|  |  | Absent | | Present | | p-value | | |
| --- | --- | --- | --- | --- | --- | --- | --- | --- |
| IFNy-Saliva | CMV viremia | 0.81 ± 0.28 | 0.76 ± 0.30 | | 0.3137 | |  |  |
|  | BKPyV viremia | 0.81 ± 0.30 | 0.70 ± 0.20 | | 0.2962 | |  |  |
|  | Anemia | 0.78 ± 0.24 | 0.80 ± 0.32 | | 0.6099 | |  |  |
|  | Diarrhea | 0.79 ± 0.30 | 0.79 ± 0.25 | | 0.8454 | |  |  |
|  | Acute rejection | 0.82 ± 0.28 | 0.72 ± 0.21 | | 0.4783 | |  |  |
|  | Chronic rejection | 0.81 ± 0.27 | — ± — | | — | |  |  |
|  | Cardiovascular events & | 0.82 ± 0.29 | 0.75 ± 0.15 | | 0.7629 | |  |  |
|  | Infectious events# | 0.77 ± 0.19 | 0.87 ± 0.37 | | 0.5398 | |  |  |
|  | Neutropenia | 0.81 ± 0.28 | 0.68 ± 0.11 | | 0.4447 | |  |  |
|  | Leucopenia | 0.75 ± 0.23 | 0.89 ± 0.39 | | 0.3854 | |  |  |
|  | Hyperglycemia | 0.79 ± 0.31 | 0.78 ± 0.23 | | 0.9062 | |  |  |
|  | Hypertension | 0.79 ± 0.22 | 0.87 ± 0.43 | | | 0.7304 | |  |
|  | Hypercholesterolemia | 0.81 ± 0.28 | 0.73 ± 0.18 | | 0.8855 | |  |  |
|  | Herpes Simplex | 0.81 ± 0.29 | 0.63 ± 0.11 | | 0.0851 | |  |  |
|  | Herpes Zoster | 0.79 ± 0.29 | 0.60 ± 0.00 | | 0.2720 | |  |  |
|  | Stomatitis | 0.79 ± 0.30 | 0.77 ± 0.24 | | 0.9298 | |  |  |
|  | Oral candidiasis | 0.78 ± 0.28 | 0.85 ± 0.33 | | 0.8599 | |  |  |
|  | Hypersensitivity | 0.78 ± 0.28 | 0.93 ± 0.58 | | 0.9784 | |  |  |
| IFNy-Serum | CMV viremia | 2.27 ± 2.09 | | 1.99 ± 1.54 | | 0.8494 | | |
|  | BKPyV viremia | 2.21 ± 1.88 | | 2.03 ± 2.12 | | 0.7225 | | |
|  | Anemia | 2.27 ± 2.12 | | 2.09 ± 1.73 | | 0.7846 | | |
|  | Diarrhea | 2.31 ± 2.06 | | 1.61 ± 0.98 | | 0.5320 | | |
|  | Acute rejection | 2.33 ± 2.09 | | 1.80 ± 2.16 | | 0.2516 | | |
|  | Chronic rejection | 2.29 ± 2.07 | | — ± — | | — | | |
|  | Cardiovascular events& | 2.53 ± 2.17 | | 1.30 ± 1.33 | | **0.0392** | | |
|  | Infectious events# | 1.71 ± 1.62 | | 3.31 ± 2.44 | | 0.0991 | | |
|  | Neutropenia | 2.38 ± 2.10 | | 0.75 ± 0.33 | | 0.1040 | | |
|  | Leucopenia | 2.28 ± 1.96 | | 1.83 ± 1.74 | | 0.2611 | | |
|  | Hyperglycemia | 2.43 ± 2.22 | | 1.66 ± 0.89 | | 0.6732 | | |
|  | Hypertension | 2.13 ± 1.87 | | 2.83 ± 2.74 | | 0.4927 | | |
|  | Hypercholesterolemia | 2.34 ± 2.14 | | 1.76 ± 1.12 | | 0.8410 | | |
|  | Herpes Simplex | 2.29 ± 1.98 | | 1.25 ± 0.59 | | 0.3380 | | |
|  | Herpes Zoster | 2.20 ± 1.92 | | 0.98 ± 0.00 | | 0.4878 | | |
|  | Stomatitis | 2.26 ± 1.95 | | 1.88 ± 1.82 | | 0.3902 | | |
|  | Oral candidiasis | 2.12 ± 1.83 | | 2.69 ± 2.83 | | 0.7799 | | |
|  | Hypersensitivity | 2.06 ± 1.78 | | 6.88 ± 0.00 | | 0.1231 | | |
| IL-10-Saliva | CMV viremia | 1.84 ± 1.09 | | 2.29 ± 2.87 | | 0.8765 | | |
|  | BKPyV viremia | 2.14 ± 2.14 | | 1.55 ± 0.74 | | 0.2515 | | |
|  | Anemia | 1.74 ± 0.61 | | 2.21 ± 2.52 | | 0.3673 | | |
|  | Diarrhea | 2.06 ± 2.10 | | 1.77 ± 0.76 | | 0.8072 | | |
|  | Acute rejection | 2.19 ± 2.21 | | 1.65 ± 0.60 | | 0.8487 | | |
|  | Chronic rejection | 2.14 ± 2.10 | | — ± — | | — | | |
|  | Cardiovascular events& | 2.20 ± 2.30 | | 1.83 ± 0.80 | | 0.7339 | | |
|  | Infectious events# | 1.77 ± 0.99 | | 2.69 ± 3.10 | | 0.8339 | | |
|  | Neutropenia | 2.19 ± 2.15 | | 1.19 ± 0.10 | | 0.1492 | | |
|  | Leucopenia | 2.11 ± 2.18 | | 1.69 ± 0.70 | | 0.9707 | | |
|  | Hyperglycemia | 1.87 ± 1.06 | | 2.27 ± 3.06 | | 0.7056 | | |
|  | Hypertension | 2.05 ± 2.24 | | 2.45 ± 1.55 | | 0.1141 | | |
|  | Hypercholesterolemia | 1.74 ± 0.75 | | 6.76 ± 6.10 | | 0.2020 | | |
|  | Herpes Simplex | 2.09 ± 2.00 | | 1.24 ± 0.23 | | 0.0719 | | |
|  | Herpes Zoster | 2.03 ± 1.93 | | 0.85 ± 0.00 | | 0.1038 | | |
|  | Stomatitis | 2.04 ± 2.16 | | 1.89 ± 0.92 | | 0.5735 | | |
|  | Oral candidiasis | 1.98 ± 1.99 | | 2.16 ± 1.18 | | 0.4062 | | |
|  | Hypersensitivity | 1.97 ± 1.93 | | 2.65 ± 1.96 | | 0.5970 | | |
| IL-10-Serum | CMV viremia | 7.09 ± 8.30 | | 14.11 ± 28.75 | | 0.5902 | | |
|  | BKPyV viremia | 10.73 ± 21.23 | | 6.30 ± 3.86 | | 0.3532 | | |
|  | Anemia | 10.17 ± 12.27 | | 9.41 ± 23.04 | | 0.2778 | | |
|  | Diarrhea | 10.48 ± 20.20 | | 6.35 ± 10.68 | | 0.1337 | | |
|  | Acute rejection | 6.53 ± 8.50 | | 6.97 ± 7.23 | | 0.8596 | | |
|  | Chronic rejection | 6.57 ± 8.31 | | — ± — | | — | | |
|  | Cardiovascular events& | 7.02 ± 8.99 | | 4.75 ± 4.73 | | 0.4703 | | |
|  | Infectious events# | 5.22 ± 6.91 | | 8.59 ± 9.99 | | 0.1134 | | |
|  | Neutropenia | 6.72 ± 8.54 | | 3.97 ± 0.14 | | 0.8589 | | |
|  | Leucopenia | 8.41 ± 10.16 | | 13.42 ± 33.14 | | 0.2480 | | |
|  | Hyperglycemia | 11.08 ± 22.33 | | 7.07 ± 8.42 | | 0.9137 | | |
|  | Hypertension | 7.42 ± 9.08 | | 3.17 ± 1.67 | | 0.1800 | | |
|  | Hypercholesterolemia | 6.49 ± 8.42 | | 7.39 ± 8.51 | | 0.9295 | | |
|  | Herpes Simplex | 10.27 ± 19.87 | | 5.56 ± 5.11 | | 0.9424 | | |
|  | Herpes Zoster | 9.88 ± 19.03 | | 3.67 ± 0.00 | | 0.8776 | | |
|  | Stomatitis | 11.40 ± 21.11 | | 3.94 ± 1.60 | | 0.6134 | | |
|  | Oral candidiasis | 10.07 ± 19.93 | | 7.16 ± 4.61 | | 0.2948 | | |
|  | Hypersensitivity | 9.83 ± 19.04 | | 5.77 ± 0.00 | | 0.5378 | | |
| IL-4-Saliva | CMV viremia | 2.12 ± 2.12 | | 1.38 ± 1.33 | | 0.0949 | | |
|  | BKPyV viremia | 2.03 ± 2.07 | | 1.22 ± 0.85 | | 0.2510 | | |
|  | Anemia | 1.88 ± 1.32 | | 1.80 ± 2.28 | | 0.2066 | | |
|  | Diarrhea | 1.95 ± 2.02 | | 1.36 ± 1.00 | | 0.4154 | | |
|  | Acute rejection | 2.01 ± 2.07 | | 1.16 ± 1.27 | | 0.1845 | | |
|  | Chronic rejection | 1.92 ± 2.00 | | — ± — | | — | | |
|  | Cardiovascular events& | 2.00 ± 2.16 | | 1.57 ± 1.08 | | 0.9069 | | |
|  | Infectious events# | 1.89 ± 2.23 | | 1.97 ± 1.69 | | 0.8642 | | |
|  | Neutropenia | 1.97 ± 2.04 | | 0.97 ± 0.00 | | 0.4179 | | |
|  | Leucopenia | 1.90 ± 2.01 | | 1.67 ± 1.47 | | 1.0000 | | |
|  | Hyperglycemia | 2.08 ± 2.16 | | 1.28 ± 0.70 | | 0.5131 | | |
|  | Hypertension | 1.53 ± 1.06 | | 3.60 ± 3.81 | | 0.2667 | | |
|  | Hypercholesterolemia | 1.73 ± 1.34 | | 4.07 ± 5.90 | | 0.8448 | | |
|  | Herpes Simplex | 1.95 ± 1.95 | | 0.94 ± 0.67 | | 0.1318 | | |
|  | Herpes Zoster | 1.87 ± 1.88 | | 0.29 ± 0.00 | | 0.1034 | | |
|  | Stomatitis | 1.97 ± 2.05 | | 1.47 ± 1.21 | | 0.5162 | | |
|  | Oral candidiasis | 1.79 ± 1.93 | | 2.26 ± 1.40 | | 0.1409 | | |
|  | Hypersensitivity | 1.81 ± 1.86 | | 2.47 ± 3.08 | | 0.8739 | | |
| IL-4-Serum | CMV viremia | 15.49 ± 20.13 | | -1.85 ± — | | 0.9341 | | |
|  | BKPyV viremia | 3.57 ± 18.00 | | 0.61 ± — | | 0.1204 | | |
|  | Anemia | 7.49 ± 19.53 | | — ± — | | 0.3623 | | |
|  | Diarrhea | 6.76 ± 16.78 | | 1.38 ± 14.45 | | 0.1399 | | |
|  | Acute rejection | 6.06 ± 19.54 | | 12.79 ± 13.72 | | 0.1280 | | |
|  | Chronic rejection | 7.18 ± 18.34 | | — ± — | | — | | |
|  | Cardiovascular events& | 9.44 ± 19.29 | | — ± — | | 0.9148 | | |
|  | Infectious events# | 9.46 ± 22.19 | | — ± — | | 0.9047 | | |
|  | Neutropenia | 8.52 ± 18.61 | | -7.53 ± — | | 0.3460 | | |
|  | Leucopenia | 12.85 ± 20.02 | | -9.41 ± 2.66 | | 0.2981 | | |
|  | Hyperglycemia | 5.48 ± 18.44 | | 0.61 ± — | | 0.6839 | | |
|  | Hypertension | 8.90 ± 22.60 | | 11.60 ± — | | 0.4757 | | |
|  | Hypercholesterolemia | 8.52 ± 18.61 | | — ± — | | 0.5142 | | |
|  | Herpes Simplex | 4.39 ± 16.36 | | — ± — | | 0.7231 | | |
|  | Herpes Zoster | 4.39 ± 16.36 | | 37.84 ± — | | 0.6950 | | |
|  | Stomatitis | 5.79 ± 18.56 | | — ± — | | 0.6702 | | |
|  | Oral candidiasis | 2.58 ± 16.04 | | — ± — | | 0.4254 | | |
|  | Hypersensitivity | 7.18 ± 18.34 | | — ± — | | 0.1788 | | |
| IL-6-Saliva | CMV viremia | 2.24 ± 2.78 | | 2.80 ± 2.47 | | 0.0651 | | |
|  | BKPyV viremia | 2.71 ± 2.83 | | 1.47 ± 1.65 | | 0.1223 | | |
|  | Anemia | 2.37 ± 2.88 | | 2.51 ± 2.50 | | 0.7331 | | |
|  | Diarrhea | 2.45 ± 2.57 | | 2.43 ± 3.16 | | 0.3606 | | |
|  | Acute rejection | 2.65 ± 2.95 | | 1.18 ± 0.22 | | 0.9598 | | |
|  | Chronic rejection | 2.49 ± 2.82 | | — ± — | | — | | |
|  | Cardiovascular events& | 2.56 ± 3.01 | | 2.18 ± 1.99 | | 0.5221 | | |
|  | Infectious events# | 2.81 ± 3.27 | | 1.92 ± 1.74 | | 0.4992 | | |
|  | Neutropenia | 2.56 ± 2.88 | | 1.23 ± 0.21 | | 0.9449 | | |
|  | Leucopenia | 2.61 ± 2.96 | | 1.97 ± 1.49 | | 0.8218 | | |
|  | Hyperglycemia | 2.36 ± 2.26 | | 2.62 ± 3.41 | | 0.5115 | | |
|  | Hypertension | 2.58 ± 3.03 | | 2.17 ± 2.05 | | 0.8490 | | |
|  | Hypercholesterolemia | 2.52 ± 2.90 | | 2.17 ± 2.05 | | 0.7311 | | |
|  | Herpes Simplex | 2.37 ± 2.55 | | 3.07 ± 3.71 | | 0.7507 | | |
|  | Herpes Zoster | 2.48 ± 2.67 | | 0.68 ± 0.00 | | 0.2137 | | |
|  | Stomatitis | 2.76 ± 2.91 | | 1.55 ± 1.49 | | 0.2708 | | |
|  | Oral candidiasis | 2.49 ± 2.69 | | 1.98 ± 2.59 | | 0.2342 | | |
|  | Hypersensitivity | 2.51 ± 2.69 | | 0.91 ± 0.33 | | 0.3456 | | |
| IL-6-Serum | CMV viremia | 3.71 ± 2.81 | | 3.46 ± 4.80 | | 0.1736 | | |
|  | BKPyV viremia | 3.45 ± 3.78 | | 4.24 ± 2.58 | | 0.0805 | | |
|  | Anemia | 3.67 ± 3.28 | | 3.58 ± 3.82 | | 0.8200 | | |
|  | Diarrhea | 3.78 ± 3.74 | | 2.88 ± 2.47 | | 0.2784 | | |
|  | Acute rejection | 3.17 ± 3.47 | | 2.98 ± 1.22 | | 0.4071 | | |
|  | Chronic rejection | 3.16 ± 3.37 | | — ± — | | — | | |
|  | Cardiovascular events& | 3.37 ± 3.64 | | 2.11 ± 1.14 | | 0.3502 | | |
|  | Infectious events# | 2.56 ± 1.79 | | 4.22 ± 5.04 | | 0.1566 | | |
|  | Neutropenia | 3.22 ± 3.47 | | 2.15 ± 0.04 | | 0.8901 | | |
|  | Leucopenia | 3.94 ± 3.93 | | 2.52 ± 1.04 | | 0.4527 | | |
|  | Hyperglycemia | 3.51 ± 2.87 | | 3.84 ± 4.72 | | 0.9712 | | |
|  | Hypertension | 3.35 ± 3.64 | | 2.39 ± 1.93 | | 0.2078 | | |
|  | Hypercholesterolemia | 2.75 ± 1.64 | | 7.73 ± 11.06 | | 0.7098 | | |
|  | Herpes Simplex | 3.71 ± 3.73 | | 2.94 ± 1.16 | | 0.7864 | | |
|  | Herpes Zoster | 3.62 ± 3.58 | | 3.87 ± 0.00 | | 0.3758 | | |
|  | Stomatitis | 3.90 ± 3.99 | | 2.76 ± 1.21 | | 0.8533 | | |
|  | Oral candidiasis | 3.63 ± 3.73 | | 3.55 ± 1.55 | | 0.3959 | | |
|  | Hypersensitivity | 3.63 ± 3.58 | | 3.37 ± 0.00 | | 0.5898 | | |
| IL-8-Saliva | CMV viremia | 107.3 ± 127.8 | | 119.6 ± 87.6 | | 0.2455 | | |
|  | BKPyV viremia | 105.7 ± 95.7 | | 135.9 ± 156.4 | | 0.7015 | | |
|  | Anemia | 116.3 ± 90.4 | | 109.2 ± 129.7 | | 0.4565 | | |
|  | Diarrhea | 110.2 ± 113.0 | | 123.6 ± 108.3 | | 0.6342 | | |
|  | Acute rejection | 107.1 ± 96.6 | | 89.1 ± 81.8 | | 0.7540 | | |
|  | Chronic rejection | 104.9 ± 93.9 | | — ± — | | — | | |
|  | Cardiovascular events& | 106.2 ± 99.7 | | 100.0 ± 75.5 | | 1.0000 | | |
|  | Infectious events# | 115.7 ± 104.6 | | 89.1 ± 76.8 | | 0.4897 | | |
|  | Neutropenia | 102.3 ± 95.2 | | 144.1 ± 83.4 | | 0.3304 | | |
|  | Leucopenia | 110.4 ± 111.4 | | 119.1 ± 115.3 | | 0.9872 | | |
|  | Hyperglycemia | 120.7 ± 122.5 | | 94.4 ± 80.5 | | 0.9636 | | |
|  | Hypertension | 115.0 ± 98.8 | | 61.0 ± 54.6 | | 0.2881 | | |
|  | Hypercholesterolemia | 108.4 ± 95.5 | | 51.9 ± 50.1 | | 0.5858 | | |
|  | Herpes Simplex | 93.1 ± 78.6 | | 283.3 ± 206.2 | | 0.0641 | | |
|  | Herpes Zoster | 112.6 ± 110.9 | | — ± — | | — | | |
|  | Stomatitis | 113.5 ± 97.3 | | 110.7 ± 141.8 | | 0.4935 | | |
|  | Oral candidiasis | 120.8 ± 114.1 | | 41.1 ± 27.4 | | 0.1385 | | |
|  | Hypersensitivity | 112.9 ± 111.7 | | 108.0 ± 134.4 | | 0.6790 | | |
| IL-8-Serum | CMV viremia | 11.94 ± 6.67 | | 11.02 ± 9.07 | | 0.4195 | | |
|  | BKPyV viremia | 11.72 ± 7.61 | | 11.32 ± 7.48 | | 0.9384 | | |
|  | Anemia | 11.84 ± 8.75 | | 11.43 ± 6.33 | | 0.7582 | | |
|  | Diarrhea | 12.03 ± 7.47 | | 9.69 ± 7.82 | | 0.1971 | | |
|  | Acute rejection | 10.29 ± 6.43 | | 9.04 ± 8.10 | | 0.8238 | | |
|  | Chronic rejection | 10.19 ± 6.46 | | — ± — | | — | | |
|  | Cardiovascular events& | 10.09 ± 6.81 | | 10.70 ± 4.64 | | 0.4216 | | |
|  | Infectious events# | 9.37 ± 5.63 | | 11.54 ± 7.66 | | 0.4154 | | |
|  | Neutropenia | 10.12 ± 6.61 | | 11.47 ± 3.59 | | 0.4201 | | |
|  | Leucopenia | 12.08 ± 7.98 | | 9.96 ± 5.44 | | 0.6603 | | |
|  | Hyperglycemia | 11.49 ± 7.73 | | 11.90 ± 7.26 | | 0.7875 | | |
|  | Hypertension | 10.68 ± 6.66 | | 8.10 ± 5.42 | | 0.3420 | | |
|  | Hypercholesterolemia | 9.88 ± 5.69 | | 13.69 ± 14.00 | | 0.9556 | | |
|  | Herpes Simplex | 11.81 ± 7.68 | | 10.05 ± 6.23 | | 0.6212 | | |
|  | Herpes Zoster | 11.50 ± 7.54 | | 17.00 ± 0.00 | | 0.2747 | | |
|  | Stomatitis | 12.66 ± 7.92 | | 8.67 ± 5.39 | | 0.1333 | | |
|  | Oral candidiasis | 11.60 ± 7.76 | | 11.77 ± 5.48 | | 0.6591 | | |
|  | Hypersensitivity | 11.92 ± 7.49 | | 5.18 ± 5.91 | | 0.1960 | | |
| TNFa-Saliva | CMV viremia | 2.32 ± 2.34 | | 3.17 ± 2.28 | | 0.2583 | | |
|  | BKPyV viremia | 2.76 ± 2.53 | | 2.19 ± 1.58 | | 0.7280 | | |
|  | Anemia | 2.68 ± 2.82 | | 2.57 ± 1.89 | | 0.6511 | | |
|  | Diarrhea | 2.59 ± 2.37 | | 2.75 ± 2.28 | | 0.9338 | | |
|  | Acute rejection | 2.55 ± 1.90 | | 2.19 ± 1.36 | | 0.9415 | | |
|  | Chronic rejection | 2.51 ± 1.84 | | — ± — | | — | | |
|  | Cardiovascular events& | 2.40 ± 1.84 | | 2.97 ± 1.90 | | 0.3040 | | |
|  | Infectious events# | 2.57 ± 1.92 | | 2.42 ± 1.77 | | 0.9138 | | |
|  | Neutropenia | 2.41 ± 1.77 | | 4.29 ± 2.91 | | 0.1902 | | |
|  | Leucopenia | 2.68 ± 2.43 | | 2.44 ± 2.13 | | 0.7261 | | |
|  | Hyperglycemia | 2.63 ± 2.47 | | 2.59 ± 2.08 | | 0.9429 | | |
|  | Hypertension | 2.44 ± 1.76 | | 2.80 ± 2.25 | | 0.8614 | | |
|  | Hypercholesterolemia | 2.53 ± 1.86 | | 2.20 ± 1.73 | | 0.8403 | | |
|  | Herpes Simplex | 2.66 ± 2.38 | | 2.28 ± 2.10 | | 0.6212 | | |
|  | Herpes Zoster | 2.67 ± 2.33 | | 0.37 ± 0.00 | | 0.0900 | | |
|  | Stomatitis | 2.86 ± 2.62 | | 1.94 ± 0.95 | | 0.9900 | | |
|  | Oral candidiasis | 2.80 ± 2.41 | | 1.18 ± 0.30 | | 0.0869 | | |
|  | Hypersensitivity | 2.69 ± 2.36 | | 1.14 ± 0.32 | | 0.2812 | | |
| TNFa-Serum | CMV viremia | 24.97 ± 21.40 | | 24.44 ± 23.64 | | 0.5798 | | |
|  | BKPyV viremia | 24.44 ± 22.00 | | 25.91 ± 23.11 | | 0.7498 | | |
|  | Anemia | 26.18 ± 25.18 | | 23.58 ± 19.38 | | 0.9752 | | |
|  | Diarrhea | 25.82 ± 23.01 | | 20.24 ± 17.42 | | 0.5973 | | |
|  | Acute rejection | 23.71 ± 20.01 | | 11.95 ± 15.98 | | 0.1280 | | |
|  | Chronic rejection | 22.47 ± 19.78 | | — ± — | | — | | |
|  | Cardiovascular events& | 24.40 ± 20.95 | | 13.94 ± 10.75 | | 0.1693 | | |
|  | Infectious events# | 22.04 ± 19.33 | | 23.14 ± 21.12 | | 0.8695 | | |
|  | Neutropenia | 22.91 ± 20.24 | | 14.58 ± 4.00 | | 0.6949 | | |
|  | Leucopenia | 26.67 ± 22.74 | | 19.10 ± 19.47 | | 0.2893 | | |
|  | Hyperglycemia | 26.16 ± 23.60 | | 21.72 ± 18.44 | | 0.7810 | | |
|  | Hypertension | 21.42 ± 17.33 | | 26.40 ± 28.32 | | 0.8579 | | |
|  | Hypercholesterolemia | 21.26 ± 18.71 | | 36.59 ± 31.05 | | 0.2671 | | |
|  | Herpes Simplex | 25.33 ± 22.76 | | 19.96 ± 14.98 | | 0.8002 | | |
|  | Herpes Zoster | 25.21 ± 22.05 | | 4.40 ± 0.00 | | 0.1592 | | |
|  | Stomatitis | 28.39 ± 23.30 | | 13.91 ± 13.10 | | **0.0351** | | |
|  | Oral candidiasis | 24.30 ± 23.05 | | 28.87 ± 9.72 | | 0.1421 | | |
|  | Hypersensitivity | 25.22 ± 22.20 | | 14.58 ± 19.93 | | 0.4701 | | |

**Supplementary Table 2:** Descriptive means and standard deviation for cytokines and clinical outcomes at T2 (*nonparametric Wilcoxon test)*

|  |  | Absent | Present | p-value |
| --- | --- | --- | --- | --- |
| IFNy-Saliva | CMV viremia | 0.79 ± 0.24 | 0.77 ± 0.18 | 0.9818 |
|  | BKPyV viremia | 0.78 ± 0.23 | 0.84 ± 0.22 | 0.6116 |
|  | Anemia | 0.76 ± 0.21 | 0.98 ± 0.37 | 0.2562 |
|  | Diarrhea | 0.80 ± 0.23 | 0.60 ± 0.06 | 0.1596 |
|  | Acute rejection | 0.77 ± 0.22 | 0.86 ± 0.29 | 0.5150 |
|  | Chronic rejection | 0.78 ± 0.23 | 0.95 ± — | 0.4149 |
|  | Cardiovascular events& | 0.78 ± 0.23 | 1.00 ± — | 0.2318 |
|  | Infectious events# | 0.80 ± 0.24 | 0.68 ± 0.07 | 0.4070 |
|  | Neutropenia | 0.79 ± 0.23 | 0.64 ± 0.06 | 0.3689 |
|  | Leucopenia | 0.78 ± 0.23 | 0.80 ± 0.26 | 0.8844 |
|  | Hyperglycemia | 0.79 ± 0.24 | 0.73 ± 0.17 | 0.7212 |
|  | Hypertension | 0.79 ± 0.23 | 0.74 ± 0.30 | 0.6674 |
|  | Hypercholesterolemia | 0.79 ± 0.23 | 0.76 ± 0.22 | 0.7252 |
|  | Herpes Simplex | 0.78 ± 0.23 | — ± — | — |
|  | Herpes Zoster | 0.79 ± 0.23 | 0.68 ± 0.00 | 0.6961 |
|  | Stomatitis | 0.78 ± 0.23 | 0.76 ± — | 0.7858 |
|  | Oral candidiasis | 0.78 ± 0.23 | — ± — | — |
|  | Hypersensitivity | 0.78 ± 0.23 | — ± — | — |
| IFNy-Serum | CMV viremia | 2.13 ± 1.99 | 1.39 ± 1.06 | 0.2911 |
|  | BKPyV viremia | 1.92 ± 1.85 | 3.04 ± — | 0.2717 |
|  | Anemia | 1.96 ± 1.85 | 1.88 ± 1.94 | 0.7707 |
|  | Diarrhea | 1.89 ± 1.90 | 2.40 ± 1.31 | 0.2217 |
|  | Acute rejection | 2.10 ± 1.92 | 1.23 ± 1.14 | 0.0619 |
|  | Chronic rejection | 2.00 ± 1.84 | 0.76 ± — | 0.2473 |
|  | Cardiovascular events& | 1.96 ± 1.83 | — ± — | — |
|  | Infectious events# | 2.07 ± 1.93 | 1.39 ± 1.11 | 0.2650 |
|  | Neutropenia | 1.99 ± 1.88 | 1.53 ± 0.88 | 0.9006 |
|  | Leucopenia | 1.71 ± 1.13 | 3.59 ± 4.17 | 0.3594 |
|  | Hyperglycemia | 1.94 ± 1.90 | 2.07 ± 1.20 | 0.7034 |
|  | Hypertension | 1.95 ± 1.93 | 1.97 ± 1.02 | 0.5824 |
|  | Hypercholesterolemia | 1.99 ± 1.88 | 1.53 ± 0.88 | 0.9006 |
|  | Herpes Simplex | 1.96 ± 1.83 | — ± — | — |
|  | Herpes Zoster | 2.04 ± 1.86 | 0.75 ± 0.02 | 0.0610 |
|  | Stomatitis | 1.95 ± 1.86 | 2.15 ± — | 0.4520 |
|  | Oral candidiasis | 1.96 ± 1.83 | — ± — | — |
|  | Hypersensitivity | 1.96 ± 1.83 | — ± — | — |
| IL-10-Saliva | CMV viremia | 1.53 ± 0.62 | 1.58 ± 0.63 | 0.7856 |
|  | BKPyV viremia | 1.55 ± 0.63 | 1.30 ± 0.16 | 0.6594 |
|  | Anemia | 1.53 ± 0.62 | 1.63 ± 0.69 | 0.6921 |
|  | Diarrhea | 1.59 ± 0.62 | 1.14 ± 0.47 | 0.1136 |
|  | Acute rejection | 1.48 ± 0.62 | 1.88 ± 0.52 | 0.1021 |
|  | Chronic rejection | 1.50 ± 0.59 | 2.66 ± — | 0.1129 |
|  | Cardiovascular events& | 1.55 ± 0.62 | 0.98 ± — | 0.2605 |
|  | Infectious events# | 1.58 ± 0.64 | 1.26 ± 0.36 | 0.3055 |
|  | Neutropenia | 1.54 ± 0.63 | 1.53 ± 0.16 | 0.6862 |
|  | Leucopenia | 1.59 ± 0.65 | 1.27 ± 0.28 | 0.3411 |
|  | Hyperglycemia | 1.52 ± 0.61 | 1.71 ± 0.71 | 0.6045 |
|  | Hypertension | 1.53 ± 0.58 | 1.58 ± 0.92 | 0.9146 |
|  | Hypercholesterolemia | 1.56 ± 0.62 | 1.13 ± 0.40 | 0.3038 |
|  | Herpes Simplex | 1.54 ± 0.61 | — ± — | — |
|  | Herpes Zoster | 1.56 ± 0.62 | 1.12 ± 0.00 | 0.2118 |
|  | Stomatitis | 1.56 ± 0.61 | 0.71 ± — | 0.1017 |
|  | Oral candidiasis | 1.54 ± 0.61 | — ± — | — |
|  | Hypersensitivity | 1.54 ± 0.61 | — ± — | — |
| IL-10 Serum | CMV viremia | 15.28 ± 24.99 | 6.32 ± 6.48 | 0.3599 |
|  | BKPyV viremia | 9.83 ± 11.94 | 114.00 ± — | 0.0942 |
|  | Anemia | 12.86 ± 22.65 | 18.32 ± 26.71 | 0.9714 |
|  | Diarrhea | 14.30 ± 24.14 | 7.94 ± 8.25 | 0.9496 |
|  | Acute rejection | 14.92 ± 24.06 | 4.11 ± 3.12 | 0.2174 |
|  | Chronic rejection | 13.42 ± 22.62 | — ± — | — |
|  | Cardiovascular events& | 12.15 ± 21.95 | 49.15 ± — | 0.1201 |
|  | Infectious events# | 14.76 ± 24.52 | 7.00 ± 7.94 | 0.2144 |
|  | Neutropenia | 13.88 ± 23.40 | 7.24 ± 2.07 | 0.5185 |
|  | Leucopenia | 13.02 ± 23.48 | 15.35 ± 20.13 | 0.8398 |
|  | Hyperglycemia | 9.03 ± 11.37 | 72.75 ± 58.34 | **0.0314** |
|  | Hypertension | 13.81 ± 23.77 | 10.07 ± 8.95 | 0.4738 |
|  | Hypercholesterolemia | 13.70 ± 22.99 | 5.77 ± — | 0.7650 |
|  | Herpes Simplex | 13.42 ± 22.62 | — ± — | — |
|  | Herpes Zoster | 14.19 ± 23.28 | 3.05 ± 0.09 | 0.4385 |
|  | Stomatitis | 13.76 ± 22.96 | 3.97 ± — | 1.0000 |
|  | Oral candidiasis | 13.42 ± 22.62 | — ± — | — |
|  | Hypersensitivity | 13.42 ± 22.62 | — ± — | — |
| IL-4-Saliva | CMV viremia | 1.86 ± 2.18 | 1.32 ± 0.44 | 0.6560 |
|  | BKPyV viremia | 1.74 ± 2.02 | 2.06 ± 1.88 | 0.6222 |
|  | Anemia | 1.58 ± 1.83 | 3.57 ± 3.03 | 0.0836 |
|  | Diarrhea | 1.83 ± 2.08 | 1.24 ± 1.04 | 0.6575 |
|  | Acute rejection | 1.60 ± 1.86 | 2.92 ± 2.79 | 0.1746 |
|  | Chronic rejection | 1.78 ± 2.01 | 1.20 ± — | 1.0000 |
|  | Cardiovascular events& | 1.77 ± 2.02 | 1.34 ± — | 0.7920 |
|  | Infectious events# | 1.63 ± 1.88 | 2.49 ± 2.61 | 0.3379 |
|  | Neutropenia | 1.78 ± 2.05 | 1.39 ± 0.59 | 0.7617 |
|  | Leucopenia | 1.89 ± 2.17 | 1.16 ± 0.42 | 0.9439 |
|  | Hyperglycemia | 1.76 ± 2.01 | 1.81 ± 2.23 | 0.8795 |
|  | Hypertension | 1.87 ± 2.07 | 0.99 ± 1.05 | 0.2924 |
|  | Hypercholesterolemia | 1.69 ± 2.02 | 2.79 ± 1.39 | 0.1114 |
|  | Herpes Simplex | 1.76 ± 1.99 | — ± — | — |
|  | Herpes Zoster | 1.78 ± 2.05 | 1.39 ± 0.59 | 0.7617 |
|  | Stomatitis | 1.78 ± 2.01 | 0.97 ± — | 0.7516 |
|  | Oral candidiasis | 1.76 ± 1.99 | — ± — | — |
|  | Hypersensitivity | 1.76 ± 1.99 | — ± — | — |
| IL-4-Serum | CMV viremia | 15.49 ± 20.13 | -1.85 ± — | 0.0872 |
|  | BKPyV viremia | 3.57 ± 18.00 | 0.61 ± — | 0.4658 |
|  | Anemia | 7.49 ± 19.53 | — ± — | 0.3072 |
|  | Diarrhea | 6.76 ± 16.78 | 1.38 ± 14.45 | 0.5163 |
|  | Acute rejection | 6.06 ± 19.54 | 12.79 ± 13.72 | 0.5163 |
|  | Chronic rejection | 7.18 ± 18.34 | — ± — | — |
|  | Cardiovascular events& | 9.44 ± 19.29 | — ± — | — |
|  | Infectious events# | 9.46 ± 22.19 | — ± — | 0.6652 |
|  | Neutropenia | 8.52 ± 18.61 | -7.53 ± — | 0.1893 |
|  | Leucopenia | 12.85 ± 20.02 | -9.41 ± 2.66 | 0.0506 |
|  | Hyperglycemia | 5.48 ± 18.44 | 0.61 ± — | 0.4658 |
|  | Hypertension | 8.90 ± 22.60 | 11.60 ± — | 0.4658 |
|  | Hypercholesterolemia | 8.52 ± 18.61 | — ± — | 0.1893 |
|  | Herpes Simplex | 4.39 ± 16.36 | — ± — | — |
|  | Herpes Zoster | 4.39 ± 16.36 | 37.84 ± — | 0.1086 |
|  | Stomatitis | 5.79 ± 18.56 | — ± — | — |
|  | Oral candidiasis | 2.58 ± 16.04 | — ± — | — |
|  | Hypersensitivity | 7.18 ± 18.34 | — ± — | — |
| IL-6-Saliva | CMV viremia | 2.23 ± 2.80 | 2.61 ± 1.45 | 0.1126 |
|  | BKPyV viremia | 2.29 ± 2.63 | 2.61 ± 0.93 | 0.2573 |
|  | Anemia | 2.22 ± 2.53 | 3.67 ± 3.50 | 0.3649 |
|  | Diarrhea | 1.94 ± 1.95 | 5.00 ± 4.81 | 0.2245 |
|  | Acute rejection | 1.92 ± 2.20 | 4.46 ± 3.55 | 0.0630 |
|  | Chronic rejection | 2.35 ± 2.59 | 1.13 ± — | 0.5992 |
|  | Cardiovascular events& | 2.31 ± 2.55 | — ± — | — |
|  | Infectious events# | 2.33 ± 2.72 | 2.17 ± 1.47 | 0.5804 |
|  | Neutropenia | 2.29 ± 2.61 | 2.68 ± 2.17 | 0.5458 |
|  | Leucopenia | 2.35 ± 2.74 | 2.06 ± 1.23 | 0.5465 |
|  | Hyperglycemia | 2.14 ± 2.29 | 4.02 ± 4.86 | 0.6837 |
|  | Hypertension | 2.04 ± 2.01 | 4.29 ± 5.10 | 0.4730 |
|  | Hypercholesterolemia | 2.40 ± 2.61 | 0.91 ± 0.33 | 0.2573 |
|  | Herpes Simplex | 2.31 ± 2.55 | — ± — | — |
|  | Herpes Zoster | 2.38 ± 2.62 | 1.22 ± 0.69 | 0.7058 |
|  | Stomatitis | 2.34 ± 2.59 | 1.19 ± — | 0.8335 |
|  | Oral candidiasis | 2.31 ± 2.55 | — ± — | — |
|  | Hypersensitivity | 2.31 ± 2.55 | — ± — | — |
| IL-6-Serum | CMV viremia | 4.29 ± 4.02 | 2.64 ± 1.46 | 0.5401 |
|  | BKPyV viremia | 3.98 ± 3.70 | 2.28 ± — | 0.8978 |
|  | Anemia | 3.85 ± 3.78 | 4.46 ± 2.83 | 0.5118 |
|  | Diarrhea | 3.91 ± 3.74 | 4.07 ± 3.17 | 0.6433 |
|  | Acute rejection | 4.19 ± 3.96 | 2.71 ± 1.36 | 0.5530 |
|  | Chronic rejection | 3.99 ± 3.70 | 2.16 ± — | 1.0000 |
|  | Cardiovascular events& | 3.79 ± 3.65 | 7.43 ± — | 0.1990 |
|  | Infectious events# | 4.08 ± 3.82 | 2.61 ± 1.41 | 0.6712 |
|  | Neutropenia | 4.02 ± 3.75 | 2.72 ± 2.15 | 0.5471 |
|  | Leucopenia | 4.07 ± 3.79 | 3.07 ± 2.93 | 0.4126 |
|  | Hyperglycemia | 4.17 ± 3.80 | 1.96 ± 0.37 | 0.4634 |
|  | Hypertension | 4.10 ± 3.74 | 1.71 ± 0.64 | 0.3083 |
|  | Hypercholesterolemia | 4.02 ± 3.68 | 1.20 ± — | 0.1232 |
|  | Herpes Simplex | 3.92 ± 3.65 | — ± — | — |
|  | Herpes Zoster | 3.93 ± 3.79 | 3.84 ± 1.26 | 0.3543 |
|  | Stomatitis | 4.01 ± 3.69 | 1.69 ± — | 0.3686 |
|  | Oral candidiasis | 3.92 ± 3.65 | — ± — | — |
|  | Hypersensitivity | 3.92 ± 3.65 | — ± — | — |
| IL-8-Saliva | CMV viremia | 159.6 ± 162.7 | 142.1 ± 142.5 | 0.5996 |
|  | BKPyV viremia | 150.0 ± 154.2 | 226.7 ± 225.2 | 0.5169 |
|  | Anemia | 151.2 ± 159.0 | 211.5 ± 136.5 | 0.3790 |
|  | Diarrhea | 118.5 ± 114.4 | 453.3 ± 126.9 | **0.0087** |
|  | Acute rejection | 135.7 ± 154.9 | 270.5 ± 118.8 | 0.0879 |
|  | Chronic rejection | 161.1 ± 156.5 | 15.6 ± — | 0.1578 |
|  | Cardiovascular events& | 157.3 ± 158.8 | 115.0 ± — | 0.8473 |
|  | Infectious events# | 137.0 ± 147.9 | 238.0 ± 181.2 | 0.3821 |
|  | Neutropenia | 149.9 ± 156.1 | 306.0 ± — | 0.3687 |
|  | Leucopenia | 159.3 ± 160.6 | 139.9 ± 149.4 | 0.8270 |
|  | Hyperglycemia | 154.1 ± 157.6 | 168.6 ± 174.4 | 0.8169 |
|  | Hypertension | 135.2 ± 120.4 | 273.8 ± 287.4 | 0.5850 |
|  | Hypercholesterolemia | 161.1 ± 156.4 | 14.9 ± — | 0.1233 |
|  | Herpes Simplex | 155.7 ± 156.0 | — ± — | — |
|  | Herpes Zoster | 165.6 ± 158.0 | 31.5 ± 15.8 | 0.1949 |
|  | Stomatitis | 158.9 ± 158.1 | 71.8 ± — | 0.7973 |
|  | Oral candidiasis | 155.7 ± 156.0 | — ± — | — |
|  | Hypersensitivity | 155.7 ± 156.0 | — ± — | — |
| IL-8-Serum | CMV viremia | 17.47 ± 20.88 | 7.34 ± 4.27 | **0.0345** |
|  | BKPyV viremia | 14.97 ± 19.59 | 20.67 ± 0.12 | **0.0592** |
|  | Anemia | 14.96 ± 19.92 | 18.90 ± 3.17 | 0.0451 |
|  | Diarrhea | 15.65 ± 19.94 | 12.00 ± 2.16 | 0.6164 |
|  | Acute rejection | 16.03 ± 20.44 | 11.36 ± 7.16 | 0.9400 |
|  | Chronic rejection | 15.65 ± 19.22 | 4.66 ± — | 0.1721 |
|  | Cardiovascular events& | 15.17 ± 19.30 | 20.04 ± — | 0.2480 |
|  | Infectious events# | 16.28 ± 20.06 | 8.33 ± 5.38 | 0.2581 |
|  | Neutropenia | 16.04 ± 19.41 | 4.17 ± 2.11 | **0.0498** |
|  | Leucopenia | 16.29 ± 20.72 | 10.94 ± 7.37 | 0.6076 |
|  | Hyperglycemia | 15.66 ± 19.85 | 11.92 ± 7.52 | 0.9501 |
|  | Hypertension | 16.17 ± 19.75 | 6.85 ± 3.68 | 0.1497 |
|  | Hypercholesterolemia | 15.64 ± 19.56 | 10.30 ± 6.55 | 0.7628 |
|  | Herpes Simplex | 15.32 ± 19.02 | — ± — | — |
|  | Herpes Zoster | 15.58 ± 19.58 | 11.22 ± 5.93 | 0.8800 |
|  | Stomatitis | 15.41 ± 19.31 | 12.32 ± — | 0.6744 |
|  | Oral candidiasis | 15.32 ± 19.02 | — ± — | — |
|  | Hypersensitivity | 15.32 ± 19.02 | — ± — | — |
| TNFa-Saliva | CMV viremia | 2.88 ± 2.88 | 3.48 ± 2.94 | 0.3596 |
|  | BKPyV viremia | 3.08 ± 2.92 | 1.80 ± 1.53 | 0.5099 |
|  | Anemia | 2.72 ± 2.67 | 5.99 ± 3.66 | 0.0834 |
|  | Diarrhea | 2.64 ± 2.32 | 5.72 ± 5.18 | 0.2848 |
|  | Acute rejection | 2.80 ± 2.77 | 4.18 ± 3.38 | 0.3070 |
|  | Chronic rejection | 3.06 ± 2.88 | 1.09 ± — | 0.3861 |
|  | Cardiovascular events& | 2.87 ± 2.79 | 7.35 ± — | 0.1686 |
|  | Infectious events# | 3.17 ± 3.05 | 2.07 ± 0.88 | 1.0000 |
|  | Neutropenia | 3.07 ± 2.93 | 2.00 ± 0.66 | 0.8836 |
|  | Leucopenia | 2.81 ± 2.77 | 3.90 ± 3.36 | 0.3781 |
|  | Hyperglycemia | 3.00 ± 2.88 | 3.02 ± 3.26 | 0.7846 |
|  | Hypertension | 2.75 ± 2.38 | 5.70 ± 6.12 | 0.3162 |
|  | Hypercholesterolemia | 3.10 ± 2.92 | 1.55 ± 0.02 | 0.4640 |
|  | Herpes Simplex | 3.01 ± 2.86 | — ± — | — |
|  | Herpes Zoster | 3.09 ± 2.92 | 1.69 ± 0.77 | 0.7418 |
|  | Stomatitis | 3.01 ± 2.90 | 2.71 ± — | 0.6463 |
|  | Oral candidiasis | 3.01 ± 2.86 | — ± — | — |
|  | Hypersensitivity | 3.01 ± 2.86 | — ± — | — |
| TNFa-Serum | CMV viremia | 25.99 ± 28.22 | 8.91 ± 4.39 | **0.0392** |
|  | BKPyV viremia | 22.54 ± 26.62 | 23.06 ± 23.89 | 1.0000 |
|  | Anemia | 17.06 ± 14.47 | 81.34 ± 52.07 | **0.0133** |
|  | Diarrhea | 22.74 ± 27.18 | 21.30 ± 19.01 | 0.9174 |
|  | Acute rejection | 19.79 ± 18.93 | 39.25 ± 52.99 | 0.5400 |
|  | Chronic rejection | 23.19 ± 26.28 | 1.61 ± — | 0.1375 |
|  | Cardiovascular events& | 20.82 ± 24.37 | 82.15 ± — | 0.1131 |
|  | Infectious events# | 22.56 ± 27.64 | 22.66 ± 16.49 | 0.4795 |
|  | Neutropenia | 23.37 ± 26.74 | 9.47 ± 0.53 | 0.2008 |
|  | Leucopenia | 22.91 ± 25.83 | 20.92 ± 30.15 | 0.2551 |
|  | Hyperglycemia | 22.91 ± 26.97 | 19.00 ± 18.19 | 0.6374 |
|  | Hypertension | 23.20 ± 26.94 | 17.68 ± 21.28 | 0.5338 |
|  | Hypercholesterolemia | 23.62 ± 26.57 | 5.33 ± 5.32 | 0.0646 |
|  | Herpes Simplex | 22.57 ± 26.15 | — ± — | — |
|  | Herpes Zoster | 23.57 ± 26.59 | 6.06 ± 6.73 | 0.1179 |
|  | Stomatitis | 22.89 ± 26.47 | 11.75 ± — | 0.7664 |
|  | Oral candidiasis | 22.57 ± 26.15 | — ± — | — |
|  | Hypersensitivity | 22.57 ± 26.15 | — ± — | — |

**Supplementary Table 3. Evolution of cytokines through the progression of patient characteristics**

| Cytokine | Characteristic | NO (T1)/NO (T@) | NO (T1)/YES (T2) | YES (T1)/NO (T2) | YES (T1)/YES (T2) | p-value |
| --- | --- | --- | --- | --- | --- | --- |
| IFNy-Saliva | CMV | -0,04 ± 0,30 | 0,08 ± 0,28 | 0,18 ± 0,26 | -0,22 ± 0,60 | 0,3700 |
|  | BKv | -0,04 ± 0,36 | -0,24 ± — | 0,04 ± 0,38 | 0,47 ± — | 0,2796 |
|  | Anemia | 0,04 ± 0,28 | -0,08 ± — | -0,12 ± 0,39 | 0,43 ± 0,27 | 0,2042 |
|  | Diarrhea | -0,02 ± 0,37 | -0,04 ± 0,11 | -0,01 ± 0,39 | — ± — | 0,9381 |
|  | Acute Rejection | -0,04 ± 0,37 | 0,06 ± 0,33 | 0,02 ± 0,44 | — ± — | 0,9931 |
|  | Chronic Rejection | -0,03 ± 0,36 | 0,27 ± — | — ± — | — ± — | 0,2548 |
|  | Cardiovascular events^&^ | -0,04 ± 0,36 | 0,24 ± — | 0,01 ± 0,37 | — ± — | 0,5811 |
|  | Infectious events^#^ | 0,02 ± 0,30 | -0,08 ± — | 0,08 ± 0,31 | -0,51 ± 0,61 | 0,2135 |
|  | Neutropenia | -0,02 ± 0,37 | -0,20 ± 0,06 | 0,20 ± 0,28 | — ± — | 0,2442 |
|  | Leucopenia | 0,02 ± 0,24 | 0,16 ± 0,31 | -0,08 ± 0,37 | -1,22 ± — | 0,2332 |
|  | Hyperglycemia | -0,04 ± 0,40 | -0,08 ± 0,23 | 0,01 ± 0,34 | 0,00 ± — | 0,9529 |
|  | Hypertension | -0,01 ± 0,29 | 0,27 ± — | -0,11 ± 0,62 | 0,00 ± — | 0,7275 |
|  | Hypercholesterolemia | -0,02 ± 0,38 | 0,21 ± — | -0,08 ± 0,11 | -0,24 ± — | 0,5041 |
|  | Herpes Simplex | -0,03 ± 0,38 | — ± — | 0,08 ± 0,10 | — ± — | 0,408 |
|  | Herpes Zoster | -0,02 ± 0,37 | 0,02 ± 0,03 | 0,04 ± — | — ± — | 0,9194 |
|  | Stomatitis | -0,08 ± 0,35 | — ± — | 0,22 ± 0,35 | 0,00 ± — | 0,1449 |
|  | Oral Candidiasis | -0,04 ± 0,35 | — ± — | 0,33 ± 0,41 | — ± — | 0,1492 |
|  | Hypersensitivity | -0,04 ± 0,35 | — ± — | 0,47 ± — | — ± — | 0,1158 |
| IFNy-Serum | CMV | -0,19 ± 3,35 | -1,27 ± 0,88 | 1,19 ± 1,67 | -1,42 ± 2,74 | 0,2193 |
|  | BKv | -0,17 ± 3,02 | 2,31 ± — | -1,31 ± 3,05 | — ± — | 0,4468 |
|  | Anemia | -0,75 ± 4,16 | -0,60 ± — | -0,01 ± 2,04 | 1,36 ± — | 0,6117 |
|  | Diarrhea | -0,43 ± 3,39 | 0,19 ± 2,74 | -0,50 ± 1,80 | 2,66 ± — | 0,5112 |
|  | Acute Rejection | -0,08 ± 2,95 | -1,77 ± 3,47 | 0,72 ± 2,53 | — ± — | 0,5442 |
|  | Chronic Rejection | -0,22 ± 3,03 | -1,89 ± — | — ± — | — ± — | 0,3862 |
|  | Cardiovascular events^&^ | -0,60 ± 3,06 | — ± — | 1,33 ± 2,13 | — ± — | 0,0796 |
|  | Infectious events^#^ | 0,35 ± 3,12 | -0,60 ± — | -1,77 ± 2,62 | -0,93 ± 2,99 | 0,3978 |
|  | Neutropenia | -0,34 ± 3,10 | -0,48 ± 2,33 | 1,64 ± — | — ± — | 0,5473 |
|  | Leucopenia | -0,77 ± 2,65 | 2,53 ± 5,73 | 0,44 ± 1,78 | -4,32 ± — | 0,3341 |
|  | Hyperglycemia | -0,13 ± 3,53 | -2,62 ± 6,97 | -0,08 ± 1,49 | -0,20 ± — | 0,9856 |
|  | Hypertension | 0,17 ± 2,94 | -2,13 ± 0,34 | -1,83 ± 3,47 | 1,74 ± 1,31 | 0,1584 |
|  | Hypercholesterolemia | -0,28 ± 3,17 | -1,01 ± — | -0,66 ± 2,08 | 1,17 ± — | 0,7903 |
|  | Herpes Simplex | -0,29 ± 3,20 | — ± — | -0,20 ± 0,98 | — ± — | 0,9514 |
|  | Herpes Zoster | -0,24 ± 3,15 | -0,86 ± 0,66 | -0,13 ± — | — ± — | 0,8013 |
|  | Stomatitis | -0,31 ± 3,29 | — ± — | -0,51 ± 1,27 | 1,64 ± — | 0,5349 |
|  | Oral Candidiasis | -0,33 ± 3,08 | — ± — | 0,49 ± 1,24 | — ± — | 0,5606 |
|  | Hypersensitivity | -0,28 ± 2,99 | — ± — | — ± — | — ± — | — |
| IL-10-Saliva | CMV | -0,22 ± 1,22 | 1,25 ± — | -0,05 ± 0,72 | -2,89 ± 4,98 | 0,1474 |
|  | BKv | -0,73 ± 2,54 | -0,37 ± — | 0,16 ± 0,51 | 0,15 ± — | 0,7656 |
|  | Anemia | -0,04 ± 0,91 | -0,15 ± — | -1,00 ± 3,05 | -0,26 ± 0,25 | 0,8998 |
|  | Diarrhea | -0,76 ± 2,58 | -0,56 ± 1,53 | 0,40 ± 0,76 | -0,64 ± — | 0,3395 |
|  | Acute Rejection | -0,21 ± 1,28 | -2,23 ± 5,24 | -0,46 ± 0,42 | — ± — | 0,3656 |
|  | Chronic Rejection | -0,59 ± 2,26 | 1,25 ± — | — ± — | — ± — | 0,0926 |
|  | Cardiovascular events^&^ | -0,66 ± 2,50 | -0,43 ± — | 0,00 ± 0,70 | — ± — | 0,7877 |
|  | Infectious events^#^ | -0,10 ± 1,22 | -0,15 ± — | -0,21 ± 0,94 | -3,57 ± 5,47 | 0,3091 |
|  | Neutropenia | -0,08 ± 0,84 | -8,06 ± 5,01 | 0,18 ± 1,03 | — ± — | 0,0609 |
|  | Leucopenia | -0,51 ± 2,80 | -1,01 ± 2,01 | -0,12 ± 0,64 | -2,39 ± — | 0,2799 |
|  | Hyperglycemia | -0,40 ± 1,40 | -0,02 ± 0,49 | -0,87 ± 3,42 | -0,15 ± — | 0,9847 |
|  | Hypertension | -0,40 ± 2,39 | -0,54 ± 2,52 | -1,50 ± 1,98 | 0,14 ± 1,10 | 0,3656 |
|  | Hypercholesterolemia | -0,07 ± 0,83 | -0,86 ± — | -5,35 ± 8,85 | -4,51 ± — | 0,2267 |
|  | Herpes Simplex | -0,60 ± 2,39 | — ± — | -0,06 ± 0,24 | — ± — | 0,9149 |
|  | Herpes Zoster | -0,58 ± 2,35 | -0,22 ± 0,11 | 0,27 ± — | — ± — | 0,8672 |
|  | Stomatitis | -0,69 ± 2,49 | — ± — | 0,15 ± 0,75 | -0,55 ± — | 0,6168 |
|  | Oral Candidiasis | -0,58 ± 2,35 | — ± — | -0,12 ± 0,15 | — ± — | 0,9758 |
|  | Hypersensitivity | -0,56 ± 2,28 | — ± — | 0,15 ± — | — ± — | 0,7213 |
| IL-10-Serum | CMV | 1,25 ± 14,74 | 0,76 ± — | 44,74 ± 46,73 | -4,76 ± 6,36 | **0,0360** |
|  | BKv | 4,30 ± 16,86 | 110,52 ± — | -4,25 ± 3,85 | — ± — | **0,0298** |
|  | Anemia | 5,88 ± 38,75 | -0,17 ± — | 4,96 ± 13,03 | 20,88 ± 33,21 | 0,5703 |
|  | Diarrhea | 8,96 ± 27,94 | -10,97 ± 21,57 | 2,97 ± 12,76 | 18,59 ± — | 0,7313 |
|  | Acute Rejection | 8,05 ± 28,37 | -2,88 ± 3,85 | 4,72 ± 12,20 | — ± — | 0,5993 |
|  | Chronic Rejection | 6,20 ± 25,11 | — ± — | — ± — | — ± — | — |
|  | Cardiovascular events^&^ | 0,49 ± 13,84 | 44,36 ± — | 24,83 ± 48,74 | — ± — | 0,1912 |
|  | Infectious events^#^ | 9,22 ± 29,28 | -0,17 ± — | 3,00 ± 24,08 | 2,09 ± 11,56 | 0,8497 |
|  | Neutropenia | 7,16 ± 26,35 | -3,25 ± 7,28 | -0,10 ± — | — ± — | 0,8903 |
|  | Leucopenia | 4,70 ± 29,04 | 7,08 ± 25,72 | 11,39 ± 14,68 | -1,52 ± — | 0,5350 |
|  | Hyperglycemia | 3,55 ± 18,38 | 110,52 ± — | -1,05 ± 8,59 | 28,57 ± — | 0,1827 |
|  | Hypertension | 8,77 ± 27,08 | -35,88 ± — | -0,37 ± 2,06 | 12,04 ± 9,26 | 0,2148 |
|  | Hypercholesterolemia | 6,95 ± 26,39 | — ± — | -1,46 ± 9,82 | 1,90 ± — | 0,7375 |
|  | Herpes Simplex | 6,29 ± 26,24 | — ± — | 5,60 ± 19,39 | — ± — | 0,8495 |
|  | Herpes Zoster | 7,33 ± 26,26 | -6,34 ± 7,46 | 1,66 ± — | — ± — | 0,4191 |
|  | Stomatitis | 7,41 ± 26,92 | — ± — | -1,83 ± 0,92 | -0,10 ± — | 0,6796 |
|  | Oral Candidiasis | 7,64 ± 26,15 | — ± — | -6,30 ± 4,71 | — ± — | 0,045 |
|  | Hypersensitivity | 6,20 ± 25,11 | — ± — | — ± — | — ± — | — |
| IL-4-Saliva | CMV | -0,13 ± 3,40 | 0,38 ± — | 1,01 ± 1,15 | -0,86 ± 2,53 | 0,3949 |
|  | BKv | -0,36 ± 2,70 | 2,24 ± — | 1,12 ± 3,86 | 0,44 ± — | 0,5344 |
|  | Anemia | 0,64 ± 2,96 | 5,46 ± — | -0,84 ± 2,65 | 0,46 ± 0,13 | 0,2492 |
|  | Diarrhea | 0,01 ± 3,31 | -0,73 ± 2,60 | 0,09 ± 0,96 | 2,01 ± — | 0,6454 |
|  | Acute Rejection | -0,23 ± 3,05 | 1,55 ± 2,62 | 0,03 ± 1,80 | — ± — | 0,5048 |
|  | Chronic Rejection | 0,00 ± 2,92 | 0,38 ± — | — ± — | — ± — | 0,7527 |
|  | Cardiovascular events^&^ | -0,15 ± 3,14 | 0,37 ± — | 0,66 ± 1,72 | — ± — | 0,4559 |
|  | Infectious events^#^ | 0,19 ± 3,18 | 5,46 ± — | -0,65 ± 1,59 | -0,68 ± 2,97 | 0,3599 |
|  | Neutropenia | 0,34 ± 2,53 | -4,54 ± 6,41 | -0,15 ± 0,21 | — ± — | 0,3461 |
|  | Leucopenia | 0,79 ± 2,71 | -2,14 ± 3,97 | 0,20 ± 1,51 | -4,92 ± — | 0,1809 |
|  | Hyperglycemia | -0,66 ± 2,77 | 2,24 ± — | 0,97 ± 3,04 | -0,92 ± — | 0,4747 |
|  | Hypertension | 0,52 ± 2,31 | -1,67 ± 2,89 | -2,77 ± 5,22 | 0,97 ± 1,48 | 0,6876 |
|  | Hypercholesterolemia | 0,25 ± 2,50 | 2,38 ± — | -0,04 ± 0,06 | -9,07 ± — | 0,1747 |
|  | Herpes Simplex | -0,04 ± 3,05 | — ± — | 0,39 ± 1,04 | — ± — | 0,4564 |
|  | Herpes Zoster | 0,00 ± 3,01 | 0,02 ± 1,56 | 0,39 ± — | — ± — | 0,9153 |
|  | Stomatitis | -0,46 ± 2,64 | — ± — | 2,05 ± 3,45 | 0,00 ± — | 0,1988 |
|  | Oral Candidiasis | 0,08 ± 3,00 | — ± — | -0,66 ± 1,06 | — ± — | 0,3805 |
|  | Hypersensitivity | 0,00 ± 2,92 | — ± — | 0,44 ± — | — ± — | 0,5994 |
| IL-4-Serum | CMV | 15,49 ± 20,13 | -1,85 ± — | 0,61 ± — | -7,01 ± 5,44 | 0,2148 |
|  | BKv | 3,57 ± 18,00 | 0,61 ± — | 19,00 ± 20,81 | — ± — | 0,5652 |
|  | Anemia | 7,49 ± 19,53 | — ± — | 3,75 ± 19,37 | 22,49 ± — | 0,4822 |
|  | Diarrhea | 6,76 ± 16,78 | 1,38 ± 14,45 | 12,02 ± 28,84 | — ± — | 0,8984 |
|  | Acute Rejection | 6,06 ± 19,54 | 12,79 ± 13,72 | — ± — | — ± — | 0,2827 |
|  | Chronic Rejection | 7,18 ± 18,34 | — ± — | — ± — | — ± — | — |
|  | Cardiovascular events ^&^ | 9,44 ± 19,29 | — ± — | -4,12 ± 6,68 | — ± — | 0,3902 |
|  | Infectious events ^#^ | 9,46 ± 22,19 | — ± — | 4,13 ± 10,56 | 1,10 ± 2,81 | 0,9762 |
|  | Neutropenia | 8,52 ± 18,61 | -7,53 ± — | — ± — | — ± — | 0,3106 |
|  | Leucopenia | 12,85 ± 20,02 | -9,41 ± 2,66 | 3,08 ± — | -0,89 ± — | 0,2699 |
|  | Hyperglycemia | 5,48 ± 18,44 | 0,61 ± — | 11,80 ± 22,45 | — ± — | 0,9689 |
|  | Hypertension | 8,90 ± 22,60 | 11,60 ± — | 3,06 ± 13,28 | — ± — | 0,7556 |
|  | Hypercholesterolemia | 8,52 ± 18,61 | — ± — | — ± — | -7,53 ± — | 0,3106 |
|  | Herpes Simplex | 4,39 ± 16,36 | — ± — | 37,84 ± — | — ± — | 0,1924 |
|  | Herpes Zoster | 4,39 ± 16,36 | 37,84 ± — | — ± — | — ± — | 0,1924 |
|  | Stomatitis | 5,79 ± 18,56 | — ± — | 22,49 ± — | — ± — | 0,3106 |
|  | Oral Candidiasis | 2,58 ± 16,04 | — ± — | 30,17 ± 10,85 | — ± — | 0,0857 |
|  | Hypersensitivity | 7,18 ± 18,34 | — ± — | — ± — | — ± — | — |
| IL-6-Saliva | CMV | 0,06 ± 3,85 | 0,95 ± 0,75 | 0,17 ± 1,63 | -2,03 ± 3,66 | 0,2640 |
|  | BKv | -0,48 ± 3,62 | -1,13 ± — | 1,08 ± 2,52 | 2,12 ± — | 0,3819 |
|  | Anemia | 0,97 ± 2,57 | -11,65 ± — | -0,79 ± 2,44 | 5,52 ± — | 0,0654 |
|  | Diarrhea | -0,43 ± 3,16 | 2,19 ± 5,65 | -0,75 ± 3,56 | 1,77 ± — | 0,6156 |
|  | Acute Rejection | -0,28 ± 2,85 | -0,59 ± 6,58 | 0,93 ± 1,19 | — ± — | 0,4369 |
|  | Chronic Rejection | -0,20 ± 3,45 | 0,42 ± — | — ± — | — ± — | 0,5286 |
|  | Cardiovascular events^&^ | -0,18 ± 3,73 | — ± — | -0,18 ± 1,26 | — ± — | 0,6076 |
|  | Infectious events^#^ | 0,00 ± 2,43 | -11,65 ± — | 0,81 ± 4,18 | -0,02 ± 1,64 | 0,3769 |
|  | Neutropenia | -0,21 ± 3,63 | -0,16 ± 0,23 | 0,17 ± 0,08 | — ± — | 0,8282 |
|  | Leucopenia | -0,45 ± 4,38 | 1,10 ± 1,13 | 0,03 ± 1,05 | -2,12 ± — | 0,2626 |
|  | Hyperglycemia | 0,07 ± 3,15 | 0,90 ± 2,86 | -0,27 ± 3,79 | -5,47 ± — | 0,4988 |
|  | Hypertension | -0,98 ± 3,17 | 4,53 ± 5,81 | 0,95 ± 2,99 | 0,96 ± 1,15 | 0,1962 |
|  | Hypercholesterolemia | -0,13 ± 3,62 | -2,10 ± — | -0,09 ± 0,33 | 0,00 ± — | 0,5984 |
|  | Herpes Simplex | 0,05 ± 3,32 | — ± — | -1,83 ± 4,04 | — ± — | 0,4733 |
|  | Herpes Zoster | 0,07 ± 3,27 | -3,95 ± 5,54 | -0,19 ± — | — ± — | 0,3706 |
|  | Stomatitis | -0,44 ± 3,61 | — ± — | 0,88 ± 2,66 | 0,11 ± — | 0,915 |
|  | Oral Candidiasis | -0,37 ± 3,35 | — ± — | 2,75 ± 3,92 | — ± — | 0,3267 |
|  | Hypersensitivity | -0,25 ± 3,43 | — ± — | 2,12 ± — | — ± — | 0,1722 |
| IL-6-Serum | CMV | 0,96 ± 3,84 | 0,39 ± 0,01 | 2,05 ± 2,96 | -3,34 ± 8,70 | 0,8343 |
|  | BKv | 0,10 ± 4,72 | 1,25 ± — | 1,27 ± 5,18 | — ± — | 0,8025 |
|  | Anemia | 1,30 ± 3,85 | 0,00 ± — | -0,50 ± 5,46 | 2,41 ± 4,14 | 0,8589 |
|  | Diarrhea | 0,11 ± 5,38 | 1,44 ± 1,41 | 1,14 ± 1,39 | — ± — | 0,2865 |
|  | Acute Rejection | 1,12 ± 3,05 | -4,09 ± 7,02 | 4,45 ± 6,89 | — ± — | 0,1014 |
|  | Chronic Rejection | 0,40 ± 4,76 | 0,39 ± — | — ± — | — ± — | 0,7974 |
|  | Cardiovascular events^&^ | -0,34 ± 4,61 | 5,33 ± — | 3,26 ± 4,09 | — ± — | 0,0535 |
|  | Infectious events^#^ | 0,74 ± 3,04 | 0,00 ± — | 2,04 ± 4,47 | -7,96 ± 11,72 | 0,4925 |
|  | Neutropenia | 1,14 ± 3,45 | -8,03 ± 11,62 | -0,43 ± — | — ± — | 0,3025 |
|  | Leucopenia | -0,29 ± 4,98 | 1,32 ± 2,68 | 2,75 ± 4,61 | — ± — | 0,6936 |
|  | Hyperglycemia | 1,46 ± 3,26 | -1,38 ± 3,71 | -0,34 ± 6,26 | -1,67 ± — | 0,2895 |
|  | Hypertension | 0,71 ± 5,21 | 0,39 ± — | -0,99 ± 2,05 | -0,43 ± — | 0,5561 |
|  | Hypercholesterolemia | 1,14 ± 3,45 | — ± — | -8,34 ± 11,19 | 0,19 ± — | 0,2434 |
|  | Herpes Simplex | 0,23 ± 5,01 | — ± — | 1,38 ± 1,73 | — ± — | 0,3059 |
|  | Herpes Zoster | 0,18 ± 4,91 | 2,09 ± 0,90 | 2,44 ± — | — ± — | 0,1801 |
|  | Stomatitis | 0,07 ± 4,62 | — ± — | 2,43 ± 5,70 | -0,43 ± — | 0,7418 |
|  | Oral Candidiasis | 0,60 ± 4,84 | — ± — | -1,16 ± 2,98 | — ± — | 0,3159 |
|  | Hypersensitivity | 0,40 ± 4,66 | — ± — | — ± — | — ± — | — |
| IL-8-Saliva | CMV | 46,7 ± 144,4 | -13,7 ± — | 76,5 ± 171,6 | -2,8 ± 222,2 | 0,7053 |
|  | BKv | 47,7 ± 172,1 | -105,5 ± — | 11,2 ± 12,1 | 183,0 ± — | 0,5658 |
|  | Anemia | 47,8 ± 157,5 | 181,0 ± — | 26,2 ± 175,7 | 65,8 ± — | 0,6684 |
|  | Diarrhea | 21,7 ± 125,0 | 295,3 ± 53,4 | -68,0 ± 127,1 | 408,2 ± — | **0,0379** |
|  | Acute Rejection | -11,7 ± 134,7 | 199,2 ± 97,9 | 146,6 ± 195,3 | — ± — | **0,0254** |
|  | Chronic Rejection | 45,2 ± 163,0 | -13,7 ± — | — ± — | — ± — | 0,5209 |
|  | Cardiovascular events^&^ | 29,1 ± 153,9 | 65,8 ± — | 85,7 ± 200,9 | — ± — | 0,8073 |
|  | Infectious events^#^ | -12,8 ± 143,4 | 181,0 ± — | 88,2 ± 126,2 | 138,8 ± 235,9 | 0,1289 |
|  | Neutropenia | 41,5 ± 165,0 | 218,7 ± — | -26,1 ± 18,2 | — ± — | 0,3249 |
|  | Leucopenia | 68,8 ± 156,8 | 34,0 ± 129,6 | 20,6 ± 191,8 | -151,9 ± — | 0,4250 |
|  | Hyperglycemia | 41,8 ± 178,3 | 105,7 ± 298,8 | 32,1 ± 114,5 | 24,2 ± — | 0,9486 |
|  | Hypertension | 12,3 ± 128,2 | 159,7 ± 245,1 | 54,6 ± 239,4 | 216,1 ± 271,6 | 0,4249 |
|  | Hypercholesterolemia | 38,3 ± 166,0 | -1,4 ± — | 121,4 ± 137,6 | — ± — | 0,5293 |
|  | Herpes Simplex | 64,3 ± 154,0 | — ± — | -127,4 ± 109,5 | — ± — | **0,0308** |
|  | Herpes Zoster | 54,9 ± 157,9 | -105,6 ± 145,4 | — ± — | — ± — | 0,1385 |
|  | Stomatitis | 44,9 ± 174,4 | — ± — | 46,5 ± 91,9 | -13,3 ± — | 0,8725 |
|  | Oral Candidiasis | 44,8 ± 163,1 | — ± — | -2,8 ± — | — ± — | 0,7001 |
|  | Hypersensitivity | 37,6 ± 160,8 | — ± — | 183,0 ± — | — ± — | 0,3044 |
| IL-8-Serum | CMV | 2,55 ± 14,92 | -1,44 ± 2,58 | 24,25 ± 31,49 | -5,05 ± 13,91 | **0,0274** |
|  | BKv | 5,30 ± 22,71 | 15,50 ± — | 0,11 ± 2,98 | 19,75 ± — | 0,2401 |
|  | Anemia | 3,14 ± 8,55 | 4,90 ± — | 6,43 ± 27,27 | 6,69 ± 6,53 | 0,531 |
|  | Diarrhea | 4,26 ± 19,90 | 1,51 ± 4,39 | 10,25 ± 26,61 | — ± — | 0,9639 |
|  | Acute Rejection | 6,38 ± 21,93 | -2,87 ± 13,76 | 7,76 ± 10,51 | — ± — | 0,4761 |
|  | Chronic Rejection | 5,36 ± 20,34 | -3,26 ± — | — ± — | — ± — | 0,3446 |
|  | Cardiovascular events^&^ | 1,98 ± 14,83 | 11,30 ± — | 20,71 ± 37,64 | — ± — | 0,1675 |
|  | Infectious events^#^ | 8,44 ± 23,79 | 4,90 ± — | 2,17 ± 9,58 | -8,34 ± 16,94 | 0,6316 |
|  | Neutropenia | 3,63 ± 13,44 | -13,64 ± 19,16 | 45,20 ± 59,12 | — ± — | 0,1042 |
|  | Leucopenia | -0,49 ± 8,77 | 3,80 ± 11,37 | 23,23 ± 36,31 | -3,47 ± — | 0,3590 |
|  | Hyperglycemia | 2,47 ± 7,57 | 7,87 ± 10,80 | 9,55 ± 30,45 | -13,52 ± — | 0,3460 |
|  | Hypertension | 6,43 ± 23,35 | 1,46 ± 6,68 | 1,96 ± 5,53 | -0,67 ± — | 0,9796 |
|  | Hypercholesterolemia | 6,33 ± 20,44 | 12,63 ± — | -13,93 ± 18,75 | -0,09 ± — | 0,2816 |
|  | Herpes Simplex | 5,54 ± 21,33 | — ± — | 1,95 ± 6,27 | — ± — | 0,956 |
|  | Herpes Zoster | 5,26 ± 21,01 | 6,57 ± 5,70 | -2,54 ± — | — ± — | 0,4517 |
|  | Stomatitis | 5,23 ± 22,33 | — ± — | 4,83 ± 9,24 | 3,39 ± — | 0,7327 |
|  | Oral Candidiasis | 5,62 ± 20,99 | — ± — | -0,04 ± 4,07 | — ± — | 0,8022 |
|  | Hypersensitivity | 4,64 ± 20,22 | — ± — | 19,75 ± — | — ± — | 0,1415 |
| TNFa-Saliva | CMV | 0,97 ± 2,67 | 0,15 ± 1,10 | -0,20 ± 2,87 | -1,02 ± 3,26 | 0,7262 |
|  | BKv | 0,33 ± 2,63 | -0,50 ± — | 0,71 ± 3,71 | 1,97 ± — | 0,7103 |
|  | Anemia | 1,08 ± 2,68 | -1,42 ± — | -0,57 ± 2,14 | 5,13 ± 3,04 | **0,0329** |
|  | Diarrhea | 0,16 ± 2,58 | 3,62 ± 4,18 | 0,06 ± 2,16 | -0,70 ± — | 0,3346 |
|  | Acute Rejection | 0,23 ± 2,77 | 1,51 ± 3,54 | 0,30 ± 1,23 | — ± — | 0,9566 |
|  | Chronic Rejection | 0,45 ± 2,77 | -0,63 ± — | — ± — | — ± — | 0,3863 |
|  | Cardiovascular events^&^ | 0,56 ± 2,77 | 2,98 ± — | -0,63 ± 2,57 | — ± — | 0,2283 |
|  | Infectious events^#^ | 0,35 ± 2,66 | -1,42 ± — | 1,69 ± 2,75 | -1,60 ± 2,36 | 0,0934 |
|  | Neutropenia | 0,65 ± 2,69 | -0,20 ± 1,06 | -2,40 ± 4,07 | — ± — | 0,521 |
|  | Leucopenia | 0,80 ± 3,01 | 1,53 ± 1,14 | -0,38 ± 2,03 | -5,04 ± — | 0,1267 |
|  | Hyperglycemia | 0,76 ± 2,71 | 0,92 ± 2,00 | -0,15 ± 3,07 | 0,00 ± — | 0,4972 |
|  | Hypertension | 0,04 ± 2,02 | 3,82 ± 6,29 | 1,07 ± 3,96 | -0,70 ± — | 0,5109 |
|  | Hypercholesterolemia | 0,47 ± 2,85 | 0,35 ± — | -0,95 ± — | 0,55 ± — | 0,7224 |
|  | Herpes Simplex | 0,56 ± 2,82 | — ± — | -0,58 ± 1,90 | — ± — | 0,5565 |
|  | Herpes Zoster | 0,55 ± 2,77 | -1,50 ± 2,73 | 0,19 ± — | — ± — | 0,6513 |
|  | Stomatitis | 0,37 ± 2,61 | — ± — | 0,67 ± 3,72 | 0,48 ± — | 0,8981 |
|  | Oral Candidiasis | 0,15 ± 2,55 | — ± — | 3,22 ± 3,60 | — ± — | 0,1215 |
|  | Hypersensitivity | 0,38 ± 2,76 | — ± — | 1,97 ± — | — ± — | 0,3329 |
| TNFa-Serum | CMV | -1,60 ± 31,03 | -1,59 ± 2,09 | 25,06 ± 23,45 | -27,72 ± 25,33 | **0,0153** |
|  | BKv | -4,77 ± 29,59 | 33,43 ± — | 14,94 ± 38,88 | 5,69 ± — | 0,3251 |
|  | Anemia | -9,67 ± 29,71 | 15,87 ± — | -0,71 ± 25,02 | 66,13 ± 31,39 | 0,0905 |
|  | Diarrhea | 2,69 ± 32,10 | -13,55 ± 36,12 | -12,43 ± 20,61 | 47,91 ± — | 0,2888 |
|  | Acute Rejection | -0,90 ± 22,84 | -6,71 ± 66,69 | 14,25 ± 22,56 | — ± — | 0,4297 |
|  | Chronic Rejection | 0,00 ± 31,82 | -0,11 ± — | — ± — | — ± — | 0,843 |
|  | Cardiovascular events^&^ | -6,29 ± 30,22 | 43,93 ± — | 22,02 ± 23,74 | — ± — | **0,0436** |
|  | Infectious events^#^ | -1,64 ± 32,70 | 15,87 ± — | 3,88 ± 26,10 | -4,09 ± 44,98 | 0,3834 |
|  | Neutropenia | 0,98 ± 30,00 | -39,27 ± 31,76 | 23,99 ± 33,93 | — ± — | 0,1149 |
|  | Leucopenia | -5,58 ± 35,44 | -0,53 ± 31,11 | 13,24 ± 20,78 | -4,95 ± — | 0,3368 |
|  | Hyperglycemia | -0,08 ± 22,78 | -22,49 ± 79,08 | 3,87 ± 37,20 | -3,84 ± — | 0,9131 |
|  | Hypertension | 1,00 ± 24,04 | -27,22 ± 38,33 | -3,16 ± 53,33 | 24,23 ± 33,49 | 0,256 |
|  | Hypercholesterolemia | 2,65 ± 31,14 | -4,07 ± — | -30,59 ± 44,04 | -16,81 ± — | 0,4467 |
|  | Herpes Simplex | 1,25 ± 32,48 | — ± — | -9,69 ± 21,21 | — ± — | 0,5003 |
|  | Herpes Zoster | 1,25 ± 31,95 | -27,26 ± 5,42 | 14,44 ± — | — ± — | 0,1356 |
|  | Stomatitis | -3,18 ± 30,54 | — ± — | 14,85 ± 36,43 | 0,00 ± — | 0,8052 |
|  | Oral Candidiasis | -2,19 ± 28,34 | — ± — | 23,36 ± 58,05 | — ± — | 0,5169 |
|  | Hypersensitivity | -0,17 ± 31,81 | — ± — | 5,69 ± — | — ± — | 0,4882 |

&**cardiovascular events**: thrombophlebitis; atherosclerosis; atherosclerosis; chest pain; deep vein thrombosis; renal artery stenosis.

#**Infectious events**: e. faecalis, pseudomonas, pneumonia, urinary tract infection - E. faecium resistant to vancomycin, staphylococcus aureus, sepsis, candida glabrata, Candida albicans, acute tubular necrosis, subcutaneous seroma, COVID, flu.
